# Accelerating the elimination of cervical cancer: cross-sectional examination of cancer prevention and control in Latin America and the Caribbean

**DOI:** 10.1016/j.lana.2026.101398

**Published:** 2026-02-04

**Authors:** Sara Benitez Majano, Nathalia Katz, Soledad Urrutia, Roberta Caixeta, Carlos Torres, Carolina Chavez Cortes, Maribel Almonte, Melissa Lopez Varon, Reina Guerrero, Kathleen Schmeler, Erin Kobetz, Corinne Ferrari, Carlos Espinal, Francisco Becerra-Posada, Karla Alfaro, Daniel Salas, Anselm Hennis, Silvana Luciani, Mauricio Maza

**Affiliations:** a Department of Noncommunicable Diseases and Mental Health, Pan American Health Organization, Washington DC, USA; b Comprehensive Immunization Special Program, Pan American Health Organization, Washington DC, USA; c Noncommunicable Diseases and Mental Health, World Health Organization, Geneva, Switzerland; d Global Oncology, Cancer Network, University of Texas MD Anderson Cancer Center, Houston, TX, USA; e Department of Gynecologic Oncology & Reproductive Medicine, MD Anderson Cancer Center, Houston, TX, USA; f Sylvester Comprehensive Cancer Center, University of Miami, Miami, FL, USA; g Global Health Department, Florida International University, Miami, FL, USA; h Basic Health International, San Salvador, El Salvador

**Keywords:** Cervical cancer, HPV vaccination, HPV screening, Cancer policy, Public health

## Abstract

**Background:**

Cervical cancer is a public health problem in Latin America and the Caribbean (LAC). The Cervical Cancer Elimination strategy sets three targets (90% HPV vaccination, 70% screening, 90% treatment) for countries to be in the path towards elimination. This study provides an overview of the current status of cervical cancer control in LAC, highlighting opportunities and challenges for cervical cancer elimination.

**Methods:**

We conducted a descriptive analysis of the cervical cancer control status in LAC, using an online questionnaire completed by delegates from health authorities of 35 countries/territories.

**Findings:**

We found marked advances in the development of national plans and cervical cancer elimination strategies, particularly in Latin America. Caribbean countries and territories face barriers in program organization and human resource provision. While HPV vaccination is systematically monitored, surveillance systems for screening and treatment are limited, reducing the ability to track program performance and progress. Transition to HPV testing is ongoing, but ensuring adequate funding and management of screen-positive females remain challenging. Gaps in histopathology and treatment —especially radiotherapy— are most pronounced in the Caribbean.

**Interpretation:**

Regional collaboration, resource mobilization, and investment in information systems and workforce capacity are essential to achieve equitable access to cervical cancer prevention and care. This analysis provides a baseline to guide future studies to support LAC countries in achieving the 90-70-90 targets.

**Funding:**

Work funded by the 10.13039/501100004892Spanish Agency for International Development Cooperation (AECID) and Gavi, the Vaccine Alliance.

**Research in context**

**Evidence before this study**

We searched PubMed and Google Scholar using the terms “cervical” AND “cancer plan” OR “cancer control” OR “screening” OR “HPV test” OR “cytology” OR “visual inspection with acetic acid” OR “colposcopy” AND “follow-up” OR “diagnos∗” OR “treatment” AND “Latin America” OR “Caribbean” OR “Americas” for publications from database inception until March 31st 2023. Previous studies have found varying levels of preparedness for cervical cancer control in Latin America and the Caribbean and highlight the need for adequate planning and implementation of national strategies that target local needs and service gaps. Improvements in the development of national cancer control plans (included in non-communicable disease plans or for specific cancers have been previously reported). The scarcity of high-quality population-based data on cancer burden to monitor implementation and impact of cancer control interventions have been previously recognized. Progress in the development of population-based cancer registries have been noted, reflecting political commitment for improving cancer control. Previously reported challenges for HPV vaccine introduction and scale-up include high prices and logistic issues for delivering vaccines to eligible individuals. For screening, though most LAC countries have long-established cytology-based screening programs, issues with low participation, generally low quality and sensitivity and inadequate follow-up of screen-positive women likely have impacted their effectiveness negatively. The availability of cancer treatment varies greatly between and within countries in the region.

**Added value of this study**

In this study, we provide an updated overview of existing facilitators, key services, opportunities and challenges in 35 countries and territories in LAC for advancing towards cervical cancer elimination. We developed and used a self-assessment tool for representatives of health authorities to track and map key activities for cervical cancer control. We found marked improvements in HPV vaccine introduction in national immunization schedules and coverages, likely related to more accessible prices, school-based delivery strategies and adoption of single-dose schedules. Though several countries have introduced HPV testing, limited/inconsistent budget to plan and deliver services, fragmented services, limited capacity to follow-up and manage screen-positive women, and the lack of monitoring systems to identify eligible individuals and reach those who require follow-up are key challenges that need to be addressed.

**Implications of all the available evidences**

The evidence presented in this study highlights the urgent need for high-quality population-based information to inform targeted health policy interventions aimed at reaching the cervical cancer elimination goals, to monitor progress, and to ensure equitable access to cervical cancer services from primary prevention to secondary and tertiary care. Innovations in the delivery of care, such as single-dose vaccine schedules, HPV-test self-sampling, screen-and-treat algorithms, and improving treatment capacity at the first level of care, as well as regional mechanisms for pooled procurement, represent crucial opportunities for the region to eliminate cervical cancer. The study's data collection instrument and findings could serve as a baseline for future investigations and for other regions committed to eliminating cervical cancer as a public health problem.

## Introduction

Cervical cancer is the third most frequent cancer and the fourth cause of cancer death in females in Latin America and the Caribbean (LAC),^1^ despite it being highly preventable through HPV vaccination, screening and treatment. In 2020, Member States adopted the Strategy to Accelerate the Elimination of Cervical Cancer as a Public Health Problem (CCEI), at the 73rd World Health Assembly, with the goal for all countries to reduce cervical cancer incidence rate to fewer than 4 cases per 100,000 females.^2^ The CCEI sets out three objectives for 2030: (i) 90% HPV vaccination coverage in girls by age 15 years; (ii) 70% screening coverage (70% of females undergoing high-performance screening tests at ages 35 and 45 years); and (iii) 90% treatment of precancerous lesions and management of 90% of invasive cancer cases.

In the Americas Region, age-standardized cervical cancer incidence is almost three times higher than the elimination target (age-standardized incidence rate, ASIR: 11.5 per 100,000 females).^3^ There are important subregional differences; North America has an ASIR of 6.4 per 100,000 females, while this rate is more than doubled (15.1 per 100,000 females) in LAC,^3^ reflecting socioeconomic inequalities and inequities in the availability and access to optimal cancer prevention and control services. LAC has the second-highest cervical cancer incidence and mortality rates globally, exceeded only by Africa. The Pan American Health Organization (PAHO) launched the Elimination Initiative in 2019 as a bold, integrated strategy to eliminate more than 30 communicable diseases and related conditions in the Americas by 2030.^4^ In this scenario, PAHO is leading the effort to make the Americas the first region in the world to eliminate cervical cancer as a public health problem.

Regarding the CCEI, many countries in LAC remain far from the vaccination, screening and treatment coverage targets. Furthermore, reliable information to monitor progress is often lacking. To support the cervical cancer elimination strategy, the Department of Non-communicable Diseases and Mental Health and the Comprehensive Immunization Program at PAHO, with support from The University of Texas MD Anderson Cancer Center, the University of Miami Sylvester Comprehensive Cancer Center and Florida International University, convened representatives from immunization and cancer programs in LAC countries to characterize their current cervical cancer prevention and control activities, including defining priority actions and national plans for the elimination of cervical cancer. This manuscript provides a summary of the progress made by LAC countries and territories towards the elimination of cervical cancer as a public health problem in the region.

## Methods

We conducted a descriptive analysis on the current regional context of cervical cancer prevention and control in LAC. Information was collected from 35 LAC countries and territories through an online questionnaire completed by delegates from health authorities. We developed the Cervical Cancer Prevention and Control Capacity Self-Assessment Tool, building on existing validated questions and/or instruments, such as the World Health Organization Country Survey of Capacity and Response to Noncommunicable Diseases (CCS),^5^ to help countries comprehensively assess their capacity for cervical cancer control (Supplementary Material). Expert reviewers were identified to review the questionnaire. They included PAHO technical advisors and professionals affiliated with partner organizations with international expertise in key areas of cancer control, such as cancer surveillance, HPV laboratory, radiotherapy, and palliative care, among others. The questionnaire was organized into five main areas: Governance and Organization, Cancer Surveillance and Information Systems, Health Promotion and Primary Prevention, Screening and Early Detection, and Capacity for Diagnosing and Treating Cervical Cancer.

Health authorities from 19 Spanish- and Portuguese-speaking countries (referred to Latin American or LA: Argentina, Bolivia, Brazil, Chile, Colombia, Costa Rica, Cuba, Dominican Republic, Ecuador El Salvador, Guatemala, Honduras, Mexico, Nicaragua, Panama, Paraguay, Peru, Uruguay, and Venezuela) and 16 English-speaking countries and territories (referred to as Caribbean or CRB: Anguilla, Antigua and Barbuda, Bahamas, Barbados, Belize, British Virgin Islands, Dominica, Grenada, Guyana, Jamaica, Montserrat, Saint Lucia, Saint Kitts and Nevis, Saint Vincent and the Grenadines, Suriname, and Trinidad and Tobago) were invited to participate in this project. Health authorities were invited to designate representatives from the vaccination and cancer programs by official communication through the PAHO country offices and Ministries of Health, or through an invitation from Florida International University to nominate delegates for their annual Global Health Conference 2023 (where the second in-person workshop took place).

Once health authorities agreed to participate and had nominated their delegates from the Cancer and Immunisation programmes, delegates from both programmes were requested to complete the self-assessment tool in an online format (using SmartSurvey) or to identify the relevant professionals to complete this self-assessment. Online meetings were held to introduce the self-assessment tool and address any questions or concerns. Delegates were invited to participate in an in-person workshop to complete and discuss the information collected and to identify priority activities needed to accelerate progress toward the elimination of cervical cancer. Participants were introduced to and provided a template document for developing a National Cervical Cancer Elimination Plan. As part of PAHO's technical cooperation, support was offered to delegates who indicated that a comprehensive national cervical cancer elimination plan was a national priority activity.

### In-person and online workshops

We organized four workshops to facilitate reviews of the status of cervical cancer programs against the global elimination targets, and to promote the development of national cervical cancer elimination plans and identify priority actions and technical cooperation for their implementation.

The first of three in-person meetings took place in Mexico City, Mexico with the support of Mexican health authorities and the PAHO country office, on August 14th and 15th, 2023. There was participation from representatives from ten Member States: Cuba, Dominican Republic, El Salvador, Guatemala, Honduras, Mexico, Nicaragua, Panama, Paraguay, and Uruguay (18 country representatives in total).

The second workshop took place in Cartagena, Colombia, during the annual Global Health Conference, organized by the Florida International University, between September 13th and 15th, 2023. In this second workshop, there was representation from seven Member States: Argentina, Bolivia, Brazil, Chile, Colombia, Ecuador and Peru (summing up to 13 country representatives).

A third workshop, held online on February 6th and 7th, 2024, aimed at Spanish-speaking countries that could not participate in the previous face-to-face workshops or did not have representation from the two technical programs that were invited (either the Cancer or the Immunization program). Countries invited to participate in this online workshop included Bolivia, Colombia, Costa Rica, Ecuador, Honduras, Peru, the Dominican Republic, and Venezuela (18 country representatives in total).

The fourth meeting took place in Miami, Florida, on the 17th and 18th of July 2024, aimed at English-speaking Caribbean countries, with the support of the University of Miami, a WHO Collaborating Centre for the elimination of cervical cancer. It had participation from Anguilla, Antigua and Barbuda, the Bahamas, Barbados, Belize, the British Virgin Islands, Dominica, Guyana, Jamaica, Montserrat, Saint Kitts and Nevis, Saint Lucia, Saint Vincent and the Grenadines, Suriname, and Trinidad and Tobago (30 country representatives in total). Delegates from Grenada were unable to attend the in-person meeting due to a national emergency; however, they participated in the online meetings and provided the requested information.

Each workshop was structured with plenary sessions by technical experts focusing on the five areas of cervical cancer control. These sessions were complemented with four main group activities. Group activities followed a general structure: participants were divided into groups with technical facilitators and discussed the five main topics of the self-assessment tool among immunization and cancer representatives. The discussions particularly focused on priority areas that need to be addressed to improve primary, secondary and tertiary prevention activities, which could eventually be collated into a national cervical cancer elimination plan.

Information on the current capacity to prevent and manage cervical cancer was collected primarily through the online questionnaire, and complemented/validated in follow-up virtual meetings. A final version of the prioritized variables reported in this manuscript was shared with country representatives for their revision and feedback via email. For HPV immunization, the information provided by country representatives was validated against the information from the WHO dashboard on HPV vaccine introduction in Member States, which summarizes official vaccine schedules and coverage data submitted regularly by Member States using the WHO/UNICEF Joint Report Form on Immunization.^6^^,^^7^

Out of the 35 countries and territories included in this project, 21 countries completed the full self-assessment questionnaire, 12 countries completed it partially, and two countries did not provide any information through the project instruments.

### Data analysis

Information collected through the online instrument, as well as Supplementary Information reported to the PAHO/WHO/UNICEF JFR (on HPV vaccination) and WHO NCD CCS 2023 (on cervical screening), was discussed and reviewed for updates with country representatives during the in-person meetings and follow-up discussions. The data collected were managed and analyzed with Microsoft Excel.

In this manuscript, we present descriptive statistics (number and percentages of LA and CRB) of characteristics and services reportedly available in the participating countries that are key to advance cervical cancer elimination in the LAC region.

### Ethics approval

No individual, de-identified patient data was used in this study.

### Role of the funding source

Funding sources had no involvement in the writing of this manuscript nor in the decision to submit it for publication.

## Results

### Governance and organization of cervical cancer control activities in Latin American and Caribbean countries

Thirty-one countries provided information on their cervical cancer control governance and program organization (17/19 LA, 89.5%; 14/16 CRB, 87.5%). In general, immunization programs run separately from cancer control programs within health authorities. Most countries (15/17 LA, 88.2%; 10/14 CRB, 71.4%) report having a Unit within the Ministry of Health responsible for cervical cancer control at the national level, either as a specific cancer control Unit or a Non-Communicable Disease (NCD) Department. However, in several of them, and depending on the available infrastructure, the responsibility of cervical cancer control falls under the responsibility of a single individual, who sometimes is also responsible for other conditions, particularly in small island developing states.

Most LA countries (14/17, 82.4%) have developed national plans for cancer control or for prioritized cancer types, including cervical cancer, as a stand-alone document or as part of an NCD control plan. Fewer CRB countries/territories (5/14, 35.7%) reported having a national cancer control plan or NCD plan that includes all or prioritized cancers. Eight LA (8/17, 47.1%) and three CRB countries (3/14, 21.4%) reported having a specific cervical cancer control program or plan with budgeted activities, while 12 (6/17, 35.3% LA; 6/14, 42.9% CRB) countries reported having one in development (Table 1).

**Table 1:** Cervical cancer control program organization and surveillance.

Country	Availability of Unit/Department within national health authority, responsible for Cervical Cancer Control	Availability of official National Cancer Control Plan	Availability of specific policy/strategy/action plans for cervical cancer control?	Availability of population- or hospital-based cancer registries
Argentina	Yes	Yes	Yes, with budgeted activities	Yes, population- and hospital-based
Bolivia	Yes	In development	In development	Yes, population-based, national
Brazil	No	Yes	Yes, with budgeted activities	Yes, population-based subnational, and hospital-based
Chile	Yes	Yes	Yes, with budgeted activities	Yes, population- and hospital-based
Colombia	Yes	Yes	In development	Yes, population-based, subnational
Costa Rica	No	Yes	No	Yes, population-based, national
Cuba	Yes	Yes	Yes, with budgeted activities	Yes, population-based, national
Dominican Republic	Yes	Yes	Yes, budget allocation pending	Yes, hospital-based
Ecuador	Yes	Yes	Yes, budget allocation pending	Yes, hospital-based
El Salvador	Yes	Yes	In development	Yes, population-based, subnational, and hospital-based
Guatemala	Yes	Yes	In development	Yes, population-based
Honduras	Yes	Yes	Yes, with budgeted activities	Yes, population- and hospital-based
Mexico	Yes	No	Yes, with budgeted activities	Yes, population-based, subnational
Panama	Yes	Yes	In development	Yes, population- and hospital-based
Paraguay	Yes	Yes	Yes, with budgeted activities	Yes, population- and hospital-based
Peru	Yes	Yes	In development	Yes, population- and hospital-based
Uruguay	Yes	No	Yes, with budgeted activities	Yes, population-based, national
CARIBBEAN				
Antigua and Barbuda	Yes	No	In development	No
Barbados	Yes	Yes	No	Yes, population-based, national
Belize	Yes	Yes	Yes, with budgeted activities	Yes, hospital-based
British Virgin Islands	No	No	In development	No
Dominica	No	Yes	No	No
Grenada	No	No	No	No
Guyana	Yes	No	Yes, with budgeted activities	Yes, population-based, national
Jamaica	Yes	Yes	In development	Yes, population- and hospital-based
Montserrat	No	No	In development	No
Saint Kitts and Nevis	Yes	No	No	No
Saint Lucia	Yes	No	In development	No
Saint Vincent and the Grenadines	Yes	No	In development	No
Suriname	Yes	Yes	Yes, budget allocation pending	No
Trinidad and Tobago	Yes	Currently under development	Yes, with budgeted activities	Yes, population-based, national

Notes: Data reported between August 2023 and July 2024 initially, validated by February 2025.

Most LA countries (15/17, 88.2%) reported having at least one population-based cancer registry (PBCR), with four countries (Bolivia, Costa Rica, Cuba, and Uruguay) reporting 100% coverage of the national population. In the Caribbean, PBCR activity has been reported in Barbados, Guyana, Jamaica, and Trinidad and Tobago (4/14, 28.8%), with the latter reporting national coverage. Ten LA countries (10/17, 58.8%) and two CRB countries (2/14, 14.3%) reported having one or several hospital-based cancer registries.

### Pilar 1: HPV vaccination

As of November 2025, all countries and territories, apart from Venezuela and Haiti (which did not participate in this exercise), have introduced the HPV vaccine in their national immunization schedule (18/19, 94.7% LA; 16/16, 100% CRB) and most of them (13/18, 72.2% LA; 14/16, 87.5% CRB) use the quadrivalent vaccine (protecting against HPV types 6, 11, 16, and 18). Cuba was the latest to introduce the (bivalent) HPV vaccine in October 2025, targeting 9-year-old girls. Fourteen (14/18, 77.7%) Spanish-speaking countries and 13 (13/16, 81.2%) Caribbean countries and territories have introduced a single-dose vaccine schedule (overall 27/34, 79.4%). Thirty countries (15/18, 83.3% LA; 15/16, 93.8% CRB) reported targeting both girls and boys (30/34; 88.2%) (Table 2).

**Table 2:** HPV immunization schedules and coverage in Latin America and the Caribbean.

Country or territory	Starting year	Type of vaccine	Target population (age in years)	Primary delivery strategy	Schedule (year of single dose introduction)	First dose coverage (%, 2023)	First dose coverage (%, 2024)
Argentina	2011	Nonavalent	Girls and boys (11)	Varies by province	1 dose (2024)	63.0	55.0
Bolivia	2017	Quadrivalent	Girls and boys (10)	School-based	1 dose (2023)	67.0	78.0
Brazil	2013	Quadrivalent	Girls and boys (9–14)	Facility-based	1 dose (2024)	87.0	79.0
Chile	2014	Nonavalent	Girls and boys (9)	School-based	1 dose (2024)	88.0	94.0
Colombia	2012	Quadrivalent	Girls and boys (9–17)	School-based	1 dose (2023)	52.0	60.0
Costa Rica	2019	Quadrivalent	Girls and boys (9–17)	School-based	2 doses	77.0	92.0
Cuba^a^	2025	Bivalent	Girls (9)	4th grade	1 dose (2025)	NA	NA
Dominican Republic	2017	Quadrivalent	Girls and boys (9–14)	Facility-based	1 dose (2024)	66.0	45.0
Ecuador	2014	Quadrivalent	Girls and boys (9–14)	School-based	1 dose (2025)	79.0	89.0
El Salvador	2020	Quadrivalent	Girls and boys (9)	School-based	1 dose (2024)	76.0	86.0
Guatemala	2018	Quadrivalent	Girls and boys (9–17)	School-based	1 dose (2023)	48.0	57.0
Honduras	2016	Quadrivalent	Girls (12–15)	School-based	1 dose (2024)	81.0	72.0
Mexico	2012	Quadrivalent	Girls and boys (9–11)	School-based	1 dose (2023)	62.0	82.0
Nicaragua	2023	Bivalent	Girls (12–14)	School-based	2 doses	78.0	NA
Panama	2008	Nonavalent	Girls and boys (10)	Mixed	2 doses	79.0	71.0
Paraguay	2012	Quadrivalent	Girls and boys (10)	Mixed	1 dose (2024)	44.0	47.0
Peru	2015	Quadrivalent	Girls and boys (9–18)	School-based	1 dose (2023)	74.0	97.0
Uruguay	2013	Quadrivalent	Girls and boys (11)	Facility-based	2 doses	67.0	77.0
Venezuela	Not yet introduced	NA	NA	NA	NA	NA	NA
**Caribbean**							
Anguilla	2016	Quadrivalent	Girls and boys (9–15)	Mixed	1 dose (2023)	12.0	18.0
Antigua and Barbuda	2018	Quadrivalent	Girls and boys (9–13)	Facility-based	2 doses	2.0	2.0
Bahamas	2015	Quadrivalent	Girls and boys (9–12)	School-based	2 doses	36.0	25.0
Barbados	2014	Quadrivalent	Girls and boys (10–11)	School-based	1 dose (2023)	50.0	43.0
Belize	2016	Quadrivalent	Girls and boys (9)	School-based	1 dose (2024)	41.0	62.0
British Virgin Islands	2019	Quadrivalent	Girls and boys (11–12)	Facility-based	1 dose	32.0	82.0
Dominica	2019	Quadrivalent	Girls and boys (10–12)	School-based	1 dose (2023)	65.0	77.0
Grenada	2019	Quadrivalent	Girls and boys (9–10)	School-based	1 dose (2023)	1.0	5.0
Guyana	2011	Quadrivalent	Girls and boys (9–16)	School-based	1 dose (2023)	40.0	71.0
Jamaica	2017	Quadrivalent	Girls and boys (11–12)	School-based	1 dose (2023)	8.0	7.0
Montserrat	2017	Nonavalent	Girls and boys (12–13)	Facility-based	1 dose	NA	NA
Saint Kitts and Nevis	2019	Quadrivalent	Girls and boys (11)	School-based	1 dose (2023)	64.0	78.0
Saint Lucia	2019	Quadrivalent	Girls and boys (11–12)	School-based	1 dose (2023)	81.0	71.0
Saint Vincent and the Grenadines	2017	Bivalent	Girls (11–12)	School-based	1 dose (2024)	0.0	8.0
Suriname	2013	Quadrivalent	Girls and boys (9–13)	School-based	1 dose (2024)	0.0	13.0
Trinidad and Tobago	2013	Quadrivalent	Girls and boys (9–14)	Mixed	2 doses	31.0	38.0

Notes: Programmatic coverages as reported on PAHO eJRF and published in WHO/UNICEF Joint Report Form on Immunization Dashboard, except for British Overseas Territories Anguilla, British Virgin Islands, and Montserrat (extracted from WHO Immunization Database). NA: not applicable.

a Cuba has introduced the bivalent HPV vaccine in October 2025, targeting 9-year-old girls. Data reported between August 2023 and July 2024 initially, validated by February 2025.

Programmatic coverage of the first dose of the HPV vaccine, as reported to the WHO/UNICEF Joint Report Form on Immunization (2024), varied widely between countries, from 45.0% to 97.0% in LA, and from 2.0% to 82.0% in CRB countries and territories. HPV vaccine coverage was considerably lower in CRB than in LA. Fourteen LA and ten CRB countries reported having nominal vaccination registration for monitoring HPV vaccine coverage.

A commonly reported challenge to improve HPV vaccination coverage was misinformation and vaccine hesitancy, particularly since the COVID-19 pandemic. That, along with widespread school closures (where the HPV vaccine is generally administered), took a significant toll on HPV vaccine coverage. All countries reported a decrease in HPV vaccine coverage since 2020, from which many are still recovering. Education and information campaigns have been available in the region; however, many country representatives report the need to involve stakeholders apart from health authorities, such as Ministries of Education, local municipalities, community leaders, and teachers in such campaigns.

### Pilar 2: screening

Ten of 19 LA countries (10/19, 52.6%) and seven CRB countries (7/16, 43.8%) reported having a cervical cancer screening program with budgeted activities (Table 3). Two LA (2/19, 10.5%) and two CRB countries (2/16, 12.5%) reported having a cervical cancer program without budgeted activities. Two LA countries reported not having a cervical screening program, but having cervical screening supported through a different organizational structure or policy document.

**Table 3:** Cervical cancer screening in Latin America and the Caribbean.

Country or territory	Existence of cervical screening program	Recommended cervical screening test	Target age range (interval)	Percentage of screening done with HPV test	Availability of nominal information recording screening activity
Argentina	Yes, budgeted	Cytology, HPV test	25–64 (3 y), 30–64 (5 y)	48.9	Yes
Bolivia	No	Cytology	25–70 (3 y)	<1.0	No
Brazil	Yes, budgeted	Cytology, PV test	25–64 (3 y)	NA	No
Chile	Yes, budgeted	Co-test cytology & HPV test	25–64 (3 y)	10.0	Yes
Colombia	No^a^	Cytology, HPV test	25–29 (3 y), 30–65 (5 y)	NA	Yes
Costa Rica	In development	Cytology, HPV test	20–29 (2 y), 30–65 (5 y)	1.3	No
Cuba	Yes, budgeted	Cytology	25–64 (3 y)	NA	No
Dominican Republic	Yes, budgeted	Cytology, HPV test	25–64 (1 y), 30–49 (5 y)	2.0	No
Ecuador	Yes, budgeted	Cytology, HPV test	<30 (3 y), 30–65 (5 y)	7.0% annual	Yes
El Salvador	Yes, not costed yet	HPV test, cytology	30–59 (5 y), <20 & >60	15.0% annual	No
Guatemala^+^	Yes, budgeted	Cytology, HPV test, VIA	25–54 (3 y), 30–39 (5 y), 40–50 (3 y)	NA	No
Honduras	In development	Cytology, VIA	All women	NA	No
Mexico	Yes, budgeted	Cytology, HPV test	25–34 (3 y), 35–64 (5 y)	66.7	Yes
Nicaragua^b^	Yes	Cytology	15–100	NA	No data
Panama	In development^a^	Cytology	21–70 (2 y)	NA	No
Paraguay	Yes, not costed yet	Cytology, HPV test	25–65 (1 y), 30–65 (5 y)	5.0	Yes
Peru	Yes, budgeted	Cytology, HPV test	25–29 & 50–54 (3 y), 30–49 (5 y)	23.0	Yes
Uruguay	Yes, budgeted	Cytology, HPV test	21–29 (3 y), 30–69 (5 y)	Starting in 2025	Yes
Venezuela^b^	Yes	Cytology	No data	No data	No data
**Caribbean**					
Anguilla	No	No data	No data	No data	No data
Antigua and Barbuda	Yes, not costed yet	HPV test	30–65 (5 y)	90.0	Under development
Bahamas	No	No data	No data	No data	No data
Barbados	Yes, budgeted	Cytology	21–65	10.0	No
Belize	Yes, budgeted	VIA, HPV test	25–65	No data	No
British Virgin Islands	No	Cytology	>25	<1.0	No
Dominica	Yes, not costed yet	Cytology	18–64	NA	No
Grenada	No	Cytology	18–65	NA	No
Guyana	Yes, budgeted	HPV test, VIA	25–49	No data	No
Jamaica	Yes, budgeted	Cytology	21–65 (1–3 y)	NA	Yes
Montserrat	In development	Cytology	No data	NA	No
Saint Kitts and Nevis	Yes, budgeted	Cytology	18–65 (3 y)	NA	No
Saint Lucia	Yes, budgeted	Cytology	18–55 (1 y)	NA	No
Saint Vincent and the Grenadines	In development	Cytology	18–60 (3 y)	NA	No
Suriname	No	VIA	Pre-menopausal 30–49	<1.0	No data
Trinidad and Tobago	Yes, budgeted	Cytology and VIA	21–65 (3 y)	NA	No

Notes: VIA: Visual inspection with acetic acid; NA: not applicable.

a There is no screening program, but a different directive supports screening.^+^: Information on cervical cancer screening reported in current national guideline.

b Information on cervical screening test and target age reported to WHO Non-Communicable Disease Country Capacity Survey, 2023. Data reported between August 2023 and July 2024 initially, validated by February 2025.

Cervical cytology was the most frequently reported test used for cervical cancer screening, and it was reportedly available as a primary screening test in all 19 LA and ten CRB countries (10/16, 62.5%) (Table 3). In 12 LA countries (12/19, 63.2%), HPV test is used as an alternative to cytology and as co-test in one country (11/19, 5.3%); HPV testing is used as the main screening in one country (1/16, 6.3%), and as an alternative to visual inspection with acetic acid (VIA) in two CRB countries (2/16, 12.5%). Self-sampling HPV testing was reportedly available in Argentina, Belize, Ecuador, El Salvador, Guatemala and Peru.

Most countries and territories offer the primary screening test upon request at the health facilities, or while eligible females attend other primary care health services. The initial screening test is generally free-at-point-of-use. Eight LA (8/19, 42.1%) and one CRB (1/16, 6.3%) country reported having nominal registers to track screening and follow-up activities; however, most reported that these systems are in development and do not yet reach adequate coverage of all screening activities, nor are they ready to report and monitor key performance indicators, such as screening coverage, positivity percentage, and proportion of females with positive screening who are followed-up and receive proper management. Countries were asked to provide the annual number of females eligible for cervical screening and the annual number of females screened for each reportedly used test. Out of the six LA countries that provided data for cytology, one-year coverage ranged from 4.0% to 32.4%; while in the six LA countries that provided information for the HPV test, one-year coverage varied from 0.4% to 48.7%. Estimated cytology one-year coverage ranged between 1.0 and 23.7% in the four CRB countries that provided information, while HPV test one-year coverage ranged between 0.5 and 4.8% (three CRB countries).

### Pilar 3. diagnosis and treatment of pre-cancer and invasive cervical cancer

Sixteen LA (16/19, 84.2%) and 15 CRB (15/16, 93.8%) countries reported information on their capacity to treat cervical pre-cancer. We asked about the availability of ablative treatment (cryotherapy and/or thermal ablation) in primary care. Nine LA (9/16, 56.3%) and five CRB countries (5/14, 35.7%) reported having thermal ablation and/or cryotherapy available for treating pre-cancer in some primary care establishments. Eleven LA (11/16, 68.8%) and nine CRB (9/15, 60.0%) countries reported having capacity for large loop excision of the transformation zone (LLETZ) in public care facilities (Table 4).

**Table 4:** Resources to diagnose and treat pre-cancerous cervical lesions and invasive cancer in Latin America and the Caribbean.

Country or territory	Treatment for pre-cancer available at screening facilities	Availability of LLETZ devices in public sector	Sufficient histopathology equipment and staff	Standardized pathology report used for cervical cancer	Availability of surgical services in public facilities	General financial cover of cancer surgery	Availability of chemotherapy services in public facilities	General financial cover of chemotherapy	Availability of radiotherapy services in public facilities	General financial cover of radiotherapy	Availability of palliative care services in public facilities^b^	General financial cover of palliative care
Argentina	No	Yes	Yes	Yes	Yes	Free	Yes	Free	Yes	Free	Yes, but insufficient (H)	Free
Bolivia	No	No data	No	No	Yes	Free	Yes	Free	Yes	Free	Yes (H)	Free
Brazil	No	No data	No	Yes	Yes	Free	Yes	Free	Yes	Free	Yes (H, OP, PC)	Free
Chile	No	Yes	No	Yes	Yes	Free	Yes	Free	Yes	Free	Yes (H, OP, PC, HC)	Free
Colombia	No	No data	No data	Yes	Yes	Free	Yes	Free	Yes	Free	Yes (H, OP, HC)	Free
Costa Rica	Yes, TA and cryo.	Yes	No data	No	Yes	Free	Yes	Free	Yes	Free	Yes (H, OP, PC, HC)	Free
Cuba	Yes, TA	Yes	Yes	Yes	Yes	Free	Yes	Free	Yes	Free	Yes (H, OP, PC, HC)	Free
Dominican Republic	No	Yes	No data	Yes	Yes	Free	Yes	Free	Yes	Free	Yes (H)	Free
Ecuador	Yes, TA	No data	No data	No data	No data	No data	No data	No data	No data	No data	No data	No data
El Salvador	Yes, TA and cryo.	Yes	Yes	No	Yes	Free	Yes	Free	Yes	Free	Yes (H, OP, PC)	Free
Honduras	Yes, TA	Yes	No	Yes	Yes	Free	Yes	Free	Outsourced to private	Out-of-pocket or co-pay	Yes (H)	Free
Mexico	Yes, cryo.	Yes	No	Yes	Yes	Free	Yes	Free	Yes	Free	Yes (H, PC, HC)	Free
Panama	No info	No data	No	Yes	Yes (social security)	Free	Yes	Free	Yes	Free	Yes (H, PC, HC)	Free (social security)
Paraguay	Limited, TA	Yes	No	Yes	Yes	Free	Yes	Free	Yes, limited	Free	Yes (H, OP, PC)	Free
Peru	Limited, TA and cryo	Yes	No	No	Yes	Free	Yes	Free	Yes	Free	Yes (H)	Free
Uruguay	Yes, cryo	Yes	Yes	Yes	Yes	Free	Yes	Free	Yes	Free	Yes (H, OP PC, HC)	Free
**Caribbean**												
Antigua and Barbuda	Yes, TA	Yes	No	No	No	Co-payment	Yes	Free	No	NA	Yes (H)	Free
Bahamas^c^	No data	No data	No	No	No data	Out-of-pocket	No data	Out-of-pocket	No data	Out-of-pocket	No data	Out-of-pocket
Barbados	No	No data	No data	No	Yes	Free	Yes	Free	External, not brachytherapy	Free	Yes (H, OP)	Free
Belize	Yes, TA and cryo.	Yes	No data	No	Yes	Free	Yes	Partly free, partly out-of-pocket	No	NA	Yes (H, OP, PC, HC)	Free
British Virgin Islands	Yes, TA	Yes	Yes	Yes	Yes	95% NHI, 5% copay	Limited	95% NHI, 5% copay	Not available in-country^a^	a	Yes (H, PC)	Out-of-pocket, charity
Dominica	No	No data	No data	Yes	Yes	Out-of-pocket	Yes	Out-of-pocket	No	NA	Yes (H, OP, PC, HC)	Out-of-pocket
Grenada	No	Yes	No	No data	Yes	Free and out-of-pocket	Yes	Out-of-pocket	No	NA	Yes (H)	Partly free/out-of-pocket
Guyana	Yes, TA and cryo.	Yes	No	Yes	Yes	Free	Yes	Free	No	NA	No	NA
Jamaica	No	Yes	No	Yes	Yes	Free	Yes	Free	Yes	Free	Yes (H, PC)	Free
Montserrat	Yes, TA and cryo.	Yes	No	No	Yes	Out-of-pocket	No	NA	No	NA	Yes, (H, OP, PC)	Out-of-pocket
Saint Kitts and Nevis	No	No	No	Yes	Yes	Out-of-pocket	Yes	Out-of-pocket	No	NA	Yes (H, HC)	Out-of-pocket
Saint Lucia	No	No data	No data	Yes	Yes	Out-of-pocket	Yes	Out-of-pocket	No	NA	Yes, (H, PC)	Out-of-pocket
Saint Vincent and the Grenadines	No	No	No	Yes	Yes	Subsidized	Yes	Free	No	NA	Yes (H, OP, HC)	Free
Suriname	No	Yes	Yes	No	Yes	Reimbursed	Yes	Reimbursed	Yes	Reimbursed	Yes (H, OP, HC)	co-pay
Trinidad and Tobago	No	Yes	No data	No data	Yes	Free	Yes	Free	Yes	Free	Yes (H, OP, PC)	Free

Notes: TA: Thermal ablation; Cryo: Cryotherapy; PHC: Primary health care; NA: Not applicable.

a Radiotherapy is available overseas, with direct costs covered 95% by the NHI and a 5% copay; indirect costs, such as travel and accommodation, are generally covered by charities.

b H: Hospital; OP: Outpatient; PC: Primary care, HC: home-based care.

c Country reports that the cost of cancer treatment is generally covered by the patient (out-of-pocket expenses). Data reported between August 2023 and July 2024 initially, validated by February 2025.

Country representatives were asked to provide a list of the main health facilities that had a pathology department available to diagnose cervical pre-cancer or cancer, and whether they considered this service to be sufficient in terms of equipment and staff (Table 4). Four LA (4/16, 25.0%) and two CRB (2/15, 13.3%) countries reported having sufficient pathology services to meet demands for cervical pre-cancer and cancer diagnosis. Eleven LA (11/16, 68.8%) and seven CRB (7/15, 46.7%) countries reported using standardized pathology reporting for cervical cancer (Table 4).

Fifteen LA (15/19, 78.9%) and 14 CRB (14/16, 87.5%) countries reported information on the availability of cervical cancer treatment. All 15 LA countries that provided such information reported having surgery and chemotherapy services available, and free-at-point-of-use at public health facilities. Radiotherapy (external beam and brachytherapy) is reportedly available free at the point of use in most LA countries, except for one (1/15, 6.7%), where it is generally outsourced to private providers, representing out-of-pocket expenses for users. Palliative care services were reported to be available to some degree in all 15 LA countries at hospital level, including as an outpatient service in eight (8/15, 53.3%), at the primary care level in nine (9/15, 60.0%), and as home-based care in seven (7/15, 46.7%) (Table 4).

Thirteen (13/14, 93.9%) CRB countries and territories reported having surgical services for cervical cancer available in the public sector and free-at-point-of-use. Chemotherapy was reported to be generally available in 12 CRB countries (12/14, 85.7%), and it was reportedly limited in the British Virgin Islands. Radiotherapy services were reportedly available and free-at-point-of-use in only four CRB countries (4/14, 28.6%). Palliative care was reportedly available (at any degree) at the hospital level in 13 CRB countries (13/14, 92.9%), as an outpatient service in seven (7/14, 50.0%), at the primary care level in seven (7/14, 50.0%), and as home-based care in five (5/14, 35.7%). When available, these services were reportedly free-at-point-of-use in six (6/13, 46.2%), while they carried out-of-pocket expenses or co-payments in seven CRB countries or territories (7/13, 53.8%).

## Discussion

We examined the status of cervical cancer prevention and control activities, in 35 countries and territories across LAC, in collaboration with representatives from health authorities. This allowed us to identify priority actions for countries to accelerate towards eliminating cervical cancer.

Having comprehensive evidence-based national cancer control plans is critical for coordinating and implementing cancer programs that include all key cancer prevention and control activities.^8^^,^^9^ Having cancer-specific plans as opposed to including cancer in non-communicable disease plans is associated with having more comprehensive plans and cancer-focused goals and targets.^9^ Our finding that many LAC countries have national cancer control plans and that fewer have cervical cancer control programs with budgeted activities, is consistent with recent literature that found that although many LAC countries have cancer control plans, several remain underfunded, potentially hindering their effectiveness.^10^

Aligned with PAHO's Plan of Action for Cervical Cancer Prevention and Control 2018–2030^11^ and the WHO Strategy to accelerate the elimination of cervical cancer, countries are increasingly developing national plans specifically to support the elimination of cervical cancer by prioritizing HPV vaccination, screening, and treatment. As a result of these workshops, several LAC countries are in the process of developing their cervical cancer elimination plans. This trend reflects the growing political commitment to tackle cervical cancer, yet, it is essential that these plans have the necessary funding and evaluation activities to facilitate adjustments and ensure their effectiveness.^10^

Key advances had been made regarding government and regional commitments such as the first Global Cervical Cancer Elimination Forum that took place in March 2024, in Cartagena, Colombia, marking a historic milestone cervical cancer control. Over 40 countries participated, and $600 million was pledged to accelerate efforts to eliminate the disease. The second Global Forum took place in Bali, Indonesia in June 2025, with several regional representatives attending and reaffirming their commitment to eliminating cervical cancer.

In the area of cancer surveillance, PBCRs, which aim to identify all cancer cases in a geographically defined population, are the gold-standard source of information for estimating cancer incidence,^12^ the main impact indicator for the cervical cancer elimination strategy. PBCRs need to have high-quality information in terms of comparability, validity, timeliness, and completeness to reliably estimate cancer incidence.^13^^,^^14^ The 2015 Commission on Cancer control in LAC reported 40% increase in cancer registries in the region between 2011 and 2014,^15^ reflecting political commitment to prioritize cancer control.^16^ The periodic publication Cancer Incidence in Five Continents (CI5, International Agency for Research on Cancer) is a compilation of the most reliable cancer incidence data, drawn from high-quality PBCRs around the world; consequently, being included in a CI5 volume represents a recognition of high-quality PBCRs and reliability of cancer incidence data. In the latest CI5 Volume (XII), only eight LA countries and one CRB country were included, to the same number included the previous volume (covering 8% of the LAC population, in contrast with 98% coverage of the North America population).^1^ In our analysis, 19 countries reported having PBCRs with national or subnational coverage, substantially more than those considered having high-quality by CI5, which highlights the need to improve and maintain data quality. Recognizing the fundamental role of PBCRs in cancer control planning and implementation, ensuring appropriate and sustained funding, and developing human resource capacity to maintain PBCRs with high-quality data remain important challenges in the region, which has been recognized in previous studies.^10^^,^^15^^,^^17^^,^^18^

LAC has seen an important increase in HPV vaccine coverage from 32% in 2012 to 70% in 2024.^6^ Previously reported barriers for its introduction, like high prices and logistical issues to deliver the vaccine^18^ have been largely tackled through regional pooled procurement and school-based delivery strategies used in most countries (Table 2). Though most countries have introduced the HPV vaccine, many are far from reaching 90% coverage, particularly in the Caribbean. Suboptimal HPV vaccine uptake was exacerbated by programmatic disruptions and supply shortages caused by the COVID-19 pandemic.^19^ The adoption of single-dose schedules, recently included in the WHO and PAHO recommendations,^20^ partly supported by regional evidence of comparable protection between one- and two-dose schedules,^21^^,^^22^ address financial and logistical challenges to HPV vaccine introduction and scale-up, and ultimately contribute to improve coverage.^19^ Several countries are closer to the 90% HPV vaccine coverage after introducing single-dose schedules. Yet, important challenges remain: addressing vaccine hesitancy and misinformation are key priority in the Caribbean, which have lower HPV vaccine coverage than LAC countries. A recent literature review and meta-analysis found 84% HPV vaccine acceptance in LAC, however, there were no included studies from English-speaking Caribbean countries.^23^ Another study examining cervical cancer stigma in the Caribbean found that cultural beliefs including fear of cancer, associations between cervical cancer and promiscuity, knowledge gaps and misinformation contribute to HPV vaccine hesitancy in the region; findings highlight the need to improve trust in healthcare systems and implementing culturally tailored to tackle stigma and misinformation in the Caribbean.^24^ Recent regional campaigns have focused on raising awareness of the safety and importance of HPV vaccination, such as the World Immunization Week 2025, which had a particular focus on making multiple educational resources supporting HPV vaccination available.^25^^,^^26^

HPV vaccine registration is substantially more established than for cervical cancer screening and treatment, with most countries having nominal vaccination registries with standardized periodic reporting nationally, and internationally to the PAHO-WHO/UNICEF Joint Report Form on immunization.^7^

Although several countries reported having information systems to record screening activity, most lack all the information to track and monitor screening effectively (e.g., screening coverage, follow-up proportion, prevalence of pre-cancer, cancer incidence, and treatment coverage). Few countries provided information on the number of females who underwent a cervical cancer screening test each year (as opposed to number of tests), which is essential (along with the number of eligible population) to estimate screening coverage reliably. This is crucial as countries transition from cytology (intervals between one and three years) to HPV testing (recommended intervals between five and ten years).^27^ Health planners and screening program managers need to know who is eligible and due for screening to increase coverage of the target population, and to prevent unnecessary testing before the five/ten year interval following a negative HPV test.

There are great challenges for having adequate monitoring to follow-up service-users through the screening and treatment pathway, for instance, identifying and linking information from multiple sources at different levels of care. All this information is key not only to improve program organization and monitor progress toward the screening and treatment coverage targets, but also to ensure that all service-users are managed adequately and in a timely manner. Electronic health records may facilitate gathering the necessary information digitally; however, most systems currently face challenges related to a lack of standardization and interoperability between different co-existing systems and fragmented services.^28^

For effective cancer surveillance, in parallel to improving PBCRs, additional efforts and resources are essential to strengthen other key information sources for tracking progress of the elimination targets: screening registries or tracking systems, and hospital-based registries or clinical audit databases to monitor treatment. Recognizing these challenges and allocating the necessary resources to tackle them is key to improving the monitoring activities for screening and treatment, and for monitoring the progress towards the 70% screening and 90% treatment targets.

Regarding the organization level of screening programs, we have refrained from using the organized versus opportunistic categorization. Rather, we considered different characteristics of cervical cancer control activities, including governance frameworks, monitoring systems, protocol and follow-up standards, among others, aligned with a recent international consensus of essential criteria for ‘organized’ cancer screening programs.^29^

Most LAC countries have long-standing cytology based screening programs with variable impact on cervical cancer burden.^18^ Low coverage, limited quality and inadequate follow-up have been identified as barriers to effective screening.^18^^,^^30^

Although WHO recommends HPV testing for cervical screening based on extensive evidence of higher sensitivity of HPV DNA testing over cytology to detect precancer and of preventing cervical cancer,^31^, ^32^, ^33^ most countries continue using cytology as their primary screening test, while introducing high-performance HPV tests. Furthermore, HPV test allows self-sampling, which is associated with higher participation.^15^ Still, the proportion of screening done with HPV test was generally low. Challenges with ensuring adequate funding to procure supplies and human resources for providing services hinder efforts to introduce and scale HPV test implementation. Other remaining challenges include the need to strengthen treatment capacity and having quality assurance mechanisms in place.^18^^,^^34^ A key opportunity for the region is the pooled procurement mechanism through PAHO's Regional Revolving Funds, which facilitates the procurement of HPV testing equipment, supplies and treatment devices at accessible prices for Member States, most of which already use this mechanism for vaccine procurement.

Few countries reported having sufficient histopathology services to meet the demands for diagnosing cervical pre-cancer and cancer. Improving capacity and quality of histopathology services is an important regional priority. Multi-step screening programs that involve colposcopy/biopsy previous to treatment require important investment and time to develop the necessary infrastructure, human resources and quality control, which are important challenges in resource-limited settings.^35^

“Screen-and-treat approaches”, which entail immediate treatment of pre-cancer and/or females with high-risk HPV genotypes, help improve treatment coverage and reduce loss to follow-up, particularly in contexts where colposcopy/biopsy services are very limited or entail long waits.^27^^,^^35^^,^^36^ With comparable clinical outcomes to cryotherapy and LLETZ,^37^^,^^38^ thermal ablation, when done using a portable device to treat pre-cancer is safe, avoids logistical challenges, such as supplying and transporting refrigerant gas needed for cryotherapy, and the need of higher specialization and anesthesia required for excisional procedures.^39^^,^^40^ Thermal ablation represents an opportunity to improve treatment capacity at primary care, reducing the burden on secondary care services and facilitating adequate follow-up of screen-positive females.

Subregional differences in cervical cancer control are particularly marked when examining countries’ resources to treat invasive cancer. In several CRB countries, surgery, chemotherapy, and palliative care services are often associated with out-of-pocket expenses, representing a barrier for effective cancer treatment. Radiotherapy services are not widely available in CRB countries, a frequent challenge of Small Island Developing States. The gaps in human resources, technology, and infrastructure for cancer treatment might require the organization of services using innovative models of care, for example, the development of regional specialized cancer centers that serve the service needs of several island states or territories. Centralization and specialization of services has been associated with improvements in cancer outcomes^41^^,^^42^; however, it is essential to balance them along with easy and timely access to service for those who need them.^43^ To ensure equitable access for potential users to be referred to these centers, it is imperative that the logistics of transportation between islands and the costs associated with accommodation for patients are considered as part of the plan.

Teamed with international collaborators and representatives from health authorities from 35 countries and territories, in this manuscript, we provide a picture of the current status of cervical cancer control in LAC, presenting recent advances, opportunities and challenges to make the pathway to eliminate cervical cancer in the region.

Given the cross-sectional and ecological perspective of this exercise, we were unable to measure changes in service provision or health outcomes within the study. Though we reflect on progress based on existing literature, definitions likely differ, limiting comparability of the findings. We hope that this exercise may serve as baseline for future analyses examining the impact of interventions on cervical cancer control, building on or adapting the proposed self-assessment tool.

This regional perspective is based on self-report, thus is susceptible to bias due to incomplete information, recall, and social desirability bias. We aimed to minimize bias by requesting reference documents and validating the provided information with respondents during follow-up correspondence and meetings to the best of our possibilities: considering the constantly evolving picture in cervical cancer elimination efforts, the reported data may not fully reflect the current situation in the region. Furthermore, our questionnaire focused on obtaining key information at national level, thus failing to reflect in-country heterogeneity in service availability, access and cancer outcomes, differences by ethnicity or in vulnerable and hard-to-reach populations. Findings represent institutional views of cervical cancer control capacity and are not statistically representative of different institutions or individuals. Despite these limitations, we reckon that these biases likely underestimate rather than overestimate the needs, highlighting that supporting countries to address resource and service gaps is essential to progress toward cervical cancer elimination in the LAC region.

The modelling study that informed the CCEI targets estimated that rapid scale-up of combined 90% coverage HPV vaccination and 70% coverage of HPV-based screening (twice per lifetime by age 35 and 45) from 2020 would result in fewer than four cases per 100,000 females by 2055–59 in very high human development (HDI) countries, approximately 20 and 50 years later in medium-HDI and low-HDI countries, respectively, adopting the same interventions.^44^ Australia, a pioneer in HPV vaccination and HPV-based screening program implementation, is on track to becoming the first country to reach elimination (fewer than 4 invasive cancers per 100,000) in the next decade if current efforts are maintained, with several high-income and high HDI countries following.^45^^,^^46^ Targeted interventions in lower-income and vulnerable populations are essential to reduce inequities and accelerate cervical cancer elimination.

### Conclusion

This assessment provides a comprehensive overview of the current landscape of cervical cancer prevention and control in LAC, highlighting notable progress in several areas, particularly the formulation of national strategies aligned with the global cervical cancer elimination strategy. However, substantial challenges persist across the continuum of care, especially in program organization, surveillance, and access to diagnosis and treatment services. Addressing these gaps—particularly in health information systems, and diagnostic and treatment resources—will be essential to improve program performance and ensure females have effective access to the services they need, thus reducing the burden of cervical cancer in the region. Strengthening national capacity, fostering regional collaboration, keeping up-to-date with novel, effective, and evidence-based strategies (e.g., transitioning to single-dose vaccine and high-performance screening tests), and mobilizing the necessary political and financial commitments will be critical steps toward achieving the cervical cancer elimination targets in the region.

## Contributors

Conceptualization: SBM, SU, SL, MM; development of data collection instruments: SBM, NK, SU, CC, RC, MV, MM; workshop facilitation: SBM, NK, SU, CT, MA, MV, RC, KS, EK, CF, KA, SL, MM; data collection and validation: SBM, NK, SU, MM; methodology: SBM, RC, CC, MM; visualization: SBM, RC, CC, MM; writing original draft: SBM, NK, SU, MM; writing, review and editing: SBM, NK, SU, RC, CT, CC, MA, MV, RG, KS, EK, CF, CE, FBP, KA, DS, AH, SL, MM. All authors have read and approved the final version of the manuscript.

## Data sharing statement

Additional data collected for this study can be shared upon reasonable request.

## Declaration of interests

All coauthors declare no conflict of interests related to the content of this manuscript.

## Appendix B Supplementary data

Translated abstract Spanish
