# Supplementary material for: Accelerating the elimination of cervical cancer: cross-sectional examination of cancer prevention and control in Latin America and the Caribbean
Source: Lancet Reg Health Am. 2026 Feb 4;55:101398. doi: 10.1016/j.lana.2026.101398 (PMC13001168; doi:10.1016/j.lana.2026.101398)
Supplement: Translated abstract Spanish [file mmc1.docx]

TLRHAMERICAS-D-25-01020R1 Translated abstract - Spanish

Disclaimer: This translation in Spanish was submitted by the authors and we reproduce it as supplied. It has not been peer reviewed. Our editorial processes have only been applied to the original abstract in English, which should serve as reference for this manuscript.

**Antecedentes**

El cáncer cervicouterino es un problema de salud pública en América Latina y el Caribe (ALC). La estrategia de Eliminación del Cáncer Cervicouterino establece tres objetivos (90% de vacunación contra el VPH, 70% de cribado, 90% de tratamiento) para que los países estén en camino hacia la eliminación. Este estudio ofrece una visión general del estado actual del control del cáncer cervicouterino en ALC, destacando oportunidades y desafíos para su eliminación.

**Métodos**

Realizamos un análisis descriptivo del estado de control del cáncer cervicouterino en ALC, utilizando un cuestionario en línea completado por delegados de autoridades sanitarias de 35 países/territorios.

**Hallazgos**

Encontramos avances notables en el desarrollo de planes nacionales y estrategias de eliminación del cáncer cervicouterino, especialmente en América Latina. Los países y territorios caribeños enfrentan barreras en la organización de programas y la provisión de recursos humanos. Aunque la vacunación contra el VPH se supervisa sistemáticamente, los sistemas de vigilancia para el tamizaje y el tratamiento son limitados, lo que reduce la capacidad de monitorear el desempeño y avances programáticos. La transición a las pruebas de VPH sigue en curso, sin embargo, garantizar una financiación adecuada y el manejo adecuado de mujeres con tamizaje positivo sigue siendo retador. Limitaciones en histopatología y tratamiento —especialmente radioterapia— son más pronunciadas en el Caribe.

**Interpretación**

La colaboración regional, la movilización de recursos y la inversión en sistemas de información y capacidad laboral son esenciales para lograr un acceso equitativo a la prevención y control del cáncer cervicouterino. Este análisis proporciona una base para guiar futuros estudios que apoyen a los países de la ALC para alcanzar los objetivos 90-70-90.

**Financiación**

Trabajo financiado por Gavi y la Agencia Española de Cooperación Internacional para el Desarrollo (AECID).

**Palabras clave**

Cáncer cervicouterino; Vacunación contra el VPH; tamizaje de VPH; política de cáncer; Salud pública.
