# Supplementary material for: Accelerating the elimination of cervical cancer: cross-sectional examination of cancer prevention and control in Latin America and the Caribbean
Source: Lancet Reg Health Am. 2026 Feb 4;55:101398. doi: 10.1016/j.lana.2026.101398 (PMC13001168; doi:10.1016/j.lana.2026.101398)
Supplement: Supplementary Material [file mmc2.docx]

Self assessment tool for cervical cancer control, PAHO/NV/NMH, last modified 3 June 2024

| **ID** | **GENERAL QUESTIONS text** | **Sub-question text** | **Answer options** | **Enable conditions** |
| --- | --- | --- | --- | --- |
|  | **Background**  PAHO is supporting the planning and implementation of several cancer control projects in the Latin America and Caribbean region (LAC) in collaboration with health authorities from Member States and PAHO country and subregional offices, as part of several global initiatives on cancer control. One of the projects is the regional implementation of the Global Strategy to accelerate the elimination of cervical cancer as a public health problem, aiming to assist Member States to increase coverage of HPV vaccination, screening and treatment (90-70-90) targets to reduce mortality from cervical cancer by 30% by 2030, accelerating the journey towards disease elimination.    This self-assessment tool is design for program managers and technical agencies to better understand the current capacity and specific needs of countries to reach the 90-70-90 cervical cancer targets, as well as for monitoring progress and evaluating policy changes. The PAHO Cancer team aims to collect and assess the current situation of cervical cancer control in countries in Latin America and the Caribbean. This information will then be used to prioritize and tailor elements of technical assistance to Member States, with a range of partner organizations, on national cancer control planning, cancer registration, cancer management, and expanding access to cancer medications through the PAHO Strategic Fund, among other areas of work.  **Purpose**  The purpose of this assessment tool is to examine built capacity and infrastructure for cervical cancer control in Member States. This information will be the basis for PAHO/WHO Country Offices, Sub-Regional Offices, Headquarters and key technical collaborators for planning future technical assistance and actions relevant to countries’ specific needs. It will serve as baseline for future monitoring of any changes in national capacity and actions for cancer control. Responses will enable regional reporting on the progress towards meeting targets set in the 2020 WHO Global strategy to accelerate the elimination of cervical cancer as a public health problem.  **Scope**  This questionnaire focuses on national or subnational (region/province/state) public health authorities and care providers. They may consist of a committee, a unit, a department or an organism. Private institutions, universities or hospital units are not within the main scope of this exercise, however some information about non-governmental stakeholders may be informative in some instances. | | | |
|  | The questionnaire covers five sections, which may be answered by the same person, work group or different collaborators. In any case, a person responsible for the handling and final submission of the questionnaire must be assigned. Questions are sequential and all of them must be answered to complete the survey. Answering each section will take approximately 45 minutes. Please note that once the questionnaire has been submitted, it is no longer possible to modify the answers. If you need to edit your answers, use the ‘save and continue later’ button. You will be asked to enter a name and email address. At the end of the questionnaire, you will have the opportunity to provide additional information or make other remarks. If you need assistance or in case of any doubts, please contact Cancer Project Coordinator Dr Sara Benitez Majano (benitezsar@paho.org). Thank you! | | | |
| Gnrl1 | Country name: |  | (Select from drop-down list) |  |
| Gnrl2 | Who is the focal point for completing this survey? |  | Q2.1. Name:  Q2.2. Position:  Q2.3. Organization:  Q2.4. Email:  Q2.5. Telephone number:  Q2.6. Address: |  |
| Gnrl3 | Sections completed by focal point: |  | Q3.1. Section I: Organization and Governance  Q3.2. Section II: Information and Surveillance  Q3.3. Section III: Primary prevention  Q3.4. Section IV: Secondary prevention  Q3.5. Section V: Diagnosis and Treatment |  |
| Gnrl4 | Please enter name, affiliation and contact information of individual(s) who completed each module, if not previously named focal point: | 1. Section I: Organization and Governance  2. Section II: Information and Surveillance  3. Section III: Primary prevention  4. Section IV: Secondary Prevention  5. Section V: Diagnosis and Treatment | 1. Name  2. Affiliation  3. Contact information |  |
| Policy, strategies, action plans and guidelines | | | | |
| Gnrl5 | Is there a Unit/Branch/Department within the Ministry of Health, or equivalent national health authority, responsible for Breast/Cervical Cancer Control at national level? | 1. Yes, specific to cervical cancer  2. Yes, as part of the Cancer or NCD Unit  3. No  4. I Don't Know | 1. Unit Name  2. Number of full-time equivalent technical staff |  |
| Gnrl6 | Does an official National Cancer Control Plan exist in your country? |  | 1. Yes, as part of NCD Control Plan  2. Yes, an NCCP for all cancer types  3. Yes, an NCCP for prioritized cancer types  4. No  77. I don’t know |  |
| Gnrl7 | Does the National Cancer Control Plan refer to any of the following? | 1. National Cervical Cancer Control Program  2. National Palliative Care Program for Cancer  3. There is no National Plan for Cancer Control in place | 1. Yes  2. No  77. I Don't Know |  |
| Gnrl8 | Are there specific policies/strategies/action plans for cervical cancer control? If so, please specify your stage of development: | 1. National Cervical Cancer Control Plan  2. National Plan for the Elimination of Cervical Cancer | 1. Yes, it is operational (with budgeted activities) [Indicate year of start of most recent version]  2. Yes, but you don't have a budget  3. Currently under development  4. There was one, but it's outdated/not in effect  5. Other Type of Plan/Strategy/Action Plan [Specify]  6. No  77. I Don't Know |  |
| Gnrl9 | Please upload any relevant policy/strategy/action plan documents for cervical cancer control |  |  |  |
| Gnrl10 | Are there national guidelines/protocols/standards for **early detection** (i.e. screening and early diagnosis) of cervical cancer? |  | 1. Yes  2. No  77. I don’t know |  |
| Gnrl11 | Are there evidence-based national clinical guidelines for the **management** of (pre-) malignant lesions of breast or cervix in your country? |  | 1. Yes  2. Currently under development  3. No  77. I don’t know |  |
| Gnrl12 | Are there clearly defined **referral systems** for further investigations and/or follow-up at next level of care for suspected cancer? |  | 1. Yes  2. Currently under development  3. No  77. I don’t know |  |
| Gnrl13 | Are there national protocols for following up patients after completion of treatment for the following cancers |  | 1. Yes  2. Currently under development  3. No  77. I don’t know |  |
| Gnrl14 | Please upload the national clinical guidelines for managing AND/OR FOLLOWING UP breast and/or cervical cancer (up to 4 files), if available | File: |  |  |
| Gnrl15 | Are there operational screening programs for cervical cancer? |  | 1. Yes (with budgeted activities, specify year of introduction)  2. Yes, still not budgeted  3. Currently under development  4. There was one but it is outdated/non effective  5. Other – please specify  6. No  77. I don’t know |  |
| Gnrl16 | Please upload the relevant policy/guideline/protocol documents supporting the screening program(s) | File: |  |  |
| Gnrl17 | Are there laws to protect patients' rights to essential treatment enforced allowing the general population to secure cancer treatment initiation and adherence? | 1. Days off work to attend cervical cancer screening and/or treatment  2. Free Cervical Cancer Screening Services Regardless of Insurance Status  3. Cervical Cancer Treatment Is Free Regardless of Insurance Status  4. Other, please specify: | 1. Yes  2. No  77. I don’t know |  |
| Gnrl18 | Are there surveillance systems in place to monitor compliance with national standards (or clinical guidelines) for the treatment of cervical cancer? | 1. Cervical Cancer Specific Surveillance  2. General Surveillance System for All/Multiple Types of Cancer | 1. Yes  2. No  77. I Don't Know |  |
| Gnrl19 | Does the country produce reports on its cervical cancer program? | 1. Cervical Cancer Specific Reports  2. General reports for all/various types of cancer, including cervical cancer | 1. Yes  2. No  77. I Don't Know |  |
| Gnrl20 | Select which cervical cancer services, if any, are included in your National Essential Health Care Package or Universal Health Coverage Priority Benefit Package: |  | 1. Screening Tests  2. Follow-up after positive screening  2. Investigation Tests for Suspected Cancer  3. Treatment of Precancerous Lesions  4. Cervical Cancer Treatment  5. Palliative and supportive care  77. I Don't Know  **[select]** |  |
| Gnrl21 | Are any of the following cancer services included in emergency (e.g. COVID-19 pandemic) response plans as essential health services? |  | 1. Screening Tests  2. Follow-up after positive screening  3. Investigation for suspected cancer  4. Treatment of pre-cancerous lesions  5. Surgical treatment of cervical cancer  6. Chemotherapy Services  7. Brachytherapy  8. External Radiation Therapy  9. Palliative care  10. Cancer services are not included in the universal coverage package  11. There is no universal coverage package  77. I Don't Know  [Comments] |  |
| Gnrl22a | Does your country have a legal and regulatory framework for radiation safety? |  | 1. Yes  2. No  77. I Don't Know |  |
| Gnrl22b | ¿Se ha actualizado el marco jurídico de acuerdo con la BSS internacional para usos médicos? | If so, has the legal framework been updated in accordance with the international BSS for medical uses?  Reference Document:  <https://www.iaea.org/publications/8930/radiation-protection-and-safety-of-radiation-sources-international-basic-safety-standards> | 1. Yes  2. No  77. I Don't Know |  |
| Gnrl23 | Has your country established, through its legal system, a radiation safety regulatory body responsible for licensing facilities and activities using radiation sources? | If yes, please indicate the regulatory body responsible for radiation safety | 1. Yes  2. No  77. I Don't Know |  |
| **Medicines and Equipment - Regulations** | | | | |
| Gnrl24 | Are there any legislative frameworks for cancer treatment, radiopharmaceutical procurement, distribution and prescription of oncology drugs? | 1. Legislative framework for vaccines  2. Legislative framework for cancer drugs  3. Legislative framework for opioid medications  4. Legislative framework for radiopharmaceuticals | 1. Supply  2. Distribution  3. Prescription  4. None  77. I don’t know [select those included in the legislative framework] |  |
| Gnrl25 | Which of the following health professionals can prescribe opioids? | 1. General Practitioners/Primary Care Physicians  2. Family/Community Physicians  3. Oncologists  4. Gynecologists  5. Medical specialists trained in opioid administration  6. Other [Please specify] | 1. Yes  2. No  77. I Don't Know |  |
| Gnrl26 | Are there barriers/restrictions to prescribing opioids? | 1. A special prescription form is required  2. Special Storage Requirements  3. Dosage Limitations  4. Cultural Barriers  5. Frequent shortages  5. Other [Please specify] | 1. Yes  2. No  77. I Don't Know |  |
| Gnrl27 | Which of the following cancer prevention vaccines are purchased by the country through PAHO's Revolving Fund? |  | 1. Hepatitis B  2. Human Papillomavirus (HPV)  3. Other (please specify):  4. Country does not use PAHO's Revolving Fund  77. I Don't Know |  |
| Gnrl28 | Is the government (i.e. the Ministry of Health) responsible for procuring and distributing radiopharmaceuticals and non-radiopharmaceuticals for the diagnosis and treatment of cancer in the public sector? |  | 1. Yes  2. No  77. I Don't Know |  |
| Gnrl29 | If not, which institution or organizations are responsible for procuring and distributing drugs and radiopharmaceuticals for the diagnosis and treatment of cancer? | 1. Drug Procurement  2. Drug Distribution  3. Drug Acquisition  4. Drug Distribution | 1. Yes  2. No  77. I Don't Know |  |
| Gnrl30 | Is there a standard process in place to ensure the consistent availability of essential medications/supplies needed for cervical cancer screening and treatment? |  | 1. Yes  2. No  77. I Don't Know |  |
| Gnrl31 | Is there a steady supply of the essential/necessary domestic cancer drugs you need for cervical cancer treatment? |  | 1. Yes  2. No  77. I Don't Know |  |
| Gnrl32 | Is there a National Drug Formulary/Essential Medicines List (EML) that includes oncology drugs? |  | 1. Yes  2. No  77. I Don't Know |  |
| Gnrl33 | Is the procurement process centralized centralization for any of the following medical supplies (medicines and/or technological devices) for cancer care for public institutions? | 1. Vaccines (e.g. Hep. B, HPV):  2. Screening technology (e.g. tomosynthesis units, PCR machines, HPV test kits):  3. Supplies for surgical procedures (e.g., thermal ablation units, biopsy supplies)  4. Chemotherapy supplies (e.g., medications, equipment)  5. Radiation therapy supplies (e.g., linear accelerator, radiation shielding materials, treatment delivery devices) | 1. Yes  2. No  77. I Don't Know |  |
| Gnrl34 | Which of the following product categories have been identified as potential items for consolidated purchase, if any: |  | 1. Vaccines (e.g. Hep. B, HPV):  2. Screening technology (e.g. tomosynthesis units, PCR machines, HPV test kits):  3. Supplies for surgical procedures (e.g., thermal ablation units, biopsy supplies)  4. Chemotherapy supplies (e.g., medications, equipment)  5. Radiation therapy supplies (e.g., linear accelerator, radiation shielding materials, treatment delivery devices) |  |
| Gnrl35 | Are there any exclusivity agreements for the sale/distribution of any of the following categories of medical products for oncology (with Pharmaceutical companies or local/regional distributors)? | 1. Vaccines (e.g. Hep. B, HPV):  2. Screening technology (e.g. tomosynthesis units, PCR machines, HPV test kits):  3. Supplies for surgical procedures (e.g. thermal ablation units, ultrasound for biopsy)  4. Supplies for chemotherapy (e.g. drugs, equipment)  5. Supplies for radiotherapy (safety and shielding, diodes, barometers, etc.) | 1. Yes  2. No  77. I Don't Know |  |
| Gnrl36 | Does the country use the PAHO Strategic Fund for any of the following oncology supplies, if so, please specify the product(s): | 1. Vaccines (e.g. Hep. B, HPV):  2. Screening technology (e.g. tomosynthesis units, PCR machines, HPV test kits):  3. Supplies for surgical procedures (e.g. thermal ablation units, ultrasound for biopsy):  4. Supplies for chemotherapy (e.g. drugs, equipment)  5. Supplies for radiotherapy (safety and shielding, diodes, barometers, etc.) |  |  |
| Gnrl37 | If yes, please specify the product(s): | 1. Immunizations (e.g., Hep. B, HPV):  2. Detection technology (e.g., PCR machines, HPV test kits):  3. Supplies for surgical procedures (e.g., thermal ablation units, biopsy supplies):  4. Chemotherapy supplies (e.g., medications, equipment)  5. Radiation therapy supplies (safety and shielding, diodes, barometers, etc.) | [select] |  |
| Gnrl38 | Would you be interested in initiating a collaboration with PAHO's Strategic Fund to help your country obtain additional oncology drugs, laboratory equipment, and testing supplies? |  | 1. Sí  2. No  77. No sé | [insert skip] |
| **Medicines and Equipment - Availability** | | | | |
| Gnrl39 | With regard to the acquisition of the following cervical cancer control devices/technologies, please select the way(s) in which they have been **funded** in your country, if available: | 1. HPV Test Kits  2. Sample Collection Kits  3. PCR Testing Machines  4. Colposcopy Devices  5. Cryotherapy Devices  6. LLETZ/LEEP Devices  7. Thermal Ablation Devices  8. Laboratory Information System | 1. Centrally funded at the national level  2. Centrally funded at the sub-national level (e.g. province, region, etc.)  3. Technology funded/donated through NGOs, aid agencies, UN agencies, or other multilateral organizations (e.g., WB, IDB)  4. The technology has been *placed* as part of the purchase agreement  5. Technology available through lease/lease  6. Funded by the public facility  7. Technology only funded and available in private facilities  8. Unfunded Technology  77. I don’t know |  |
| Gnrl40 | For the following devices/technologies and/or cervical cancer control, please select the form(s) in which they have been **purchased** in your country, if available: | 1. HPV Test Kits  2. Sample Collection Kits  3. PCR Testing Machines  4. Colposcopy Devices  5. Cryotherapy Devices  6. LLETZ/LEEP Devices  7. Thermal Ablation Devices  8. Laboratory Information System | 1. Direct Supplier Procurement  2. Local Distributor  3. PAHO Strategic Fund  4. Other Regional Procurement Agent (Name)  5. Global Purchasing Agent (name)  6. Other [Specify]  77. I Don't Know |  |
| Gnrl41 | Which of the following **pathology** tests* and/or services are generally available in the public sector?  ** Generally available: reaches 50% or more of patients in need*  *Generally unavailable: reaches less than 50% of patients in need* | 1. Immunohistochemical staining  2. Pathological Processing of Specimens (Slides)  3. Slide scanner (for telepathology)  4. Cytogenetics (conventional)  5. Cytogenetics (molecular)  6. Blood Films  7. Bone marrow morphology  8. CSF 'cytospin' (cytocentrifugation) | 1. Yes  2. No, only in private  3. Not available in the country  77. I Don't Know |  |
| Gnrl42 | From the following devices/technologies, select those whose acquisition was adversely impacted due to the COVID-19 pandemic: |  | 1. PCR Testing Machine  2. HPV Test Kits  3. Colposcopy Devices  4. Cryotherapy Devices  5. LLETZ Devices  6. Thermal Ablation Devices  7. None were affected due to the COVID-19 pandemic  77. I Don't Know  **[Select]** |  |
| Gnrl43 | Describe the availability* of the following essential medications and test items in the past 3 months:  ** Generally available: reaches 50% or more of patients in need*  *Generally unavailable: reaches less than 50% of patients in need* | 1. Non-opioid pain relievers (e.g., acetaminophen, ibuprofen, others)  2. Opioid analgesics (e.g., acetaminophen, ibuprofen, others)  3. Antibiotics for the treatment of cervicitis and sexually transmitted infections (STIs) according to national guidelines  4. HPV specimen collection tubes and/or test kits and cartridges (e.g., GeneXpert)  5. HIV Test Kits  6. Pregnancy Tests  7. Chemotherapy Drugs for Cervical Cancer Treatment | 1. Generally available for purchase, copay, or subsidized at public outpatient pharmacies/clinics,  2. Generally available for purchase, copay, or subsidy at private outpatient pharmacies/clinics.  3. Dispensed in public hospitals or inpatient centers  4. Dispensed in private hospitals or inpatient centers  5. Unavailable due to product or ingredient shortages  77. I Don't Know |  |
| Gnrl44 | If essential oncology drugs are sometimes generally not available*, what are the main causes of this? (Check all that apply)  ** Generally available: reaches 50% or more of patients in need*  *Generally unavailable: reaches less than 50% of patients in need* |  | 1. Not on the procurement list (facility or domestic)  2. Lack of financial resources for purchase (installation or domestic)  3. Stock-outs/inaccurate forecasts  4. Challenges with Supply Chain Management  5. Available on-premise but unaffordable for patients  6. Global Scarcity  7. Other (please specify):  77. I Don't Know  **[Select]** |  |
| Gnrl45 | How often does your country run out of cervical cancer drugs? |  | 1. Never 0%  2. Rarely (<1%)  3. Sometimes (2-25%)  4. Mostly (>25%)  5. Always (100%)  77. I Don't Know |  |
| Gnrl46 | How often does your country run out of pain management drugs? |  | 1. Never 0%  2. Rarely (<1%)  3. Sometimes (2-25%)  4. Mostly (>25%)  5. Always (100%)  77. I Don't Know |  |
| Gnrl47 | Have studies been conducted on the prevalence of substandard and falsified cancer drugs used for cervical cancer in your country? |  | 1. Yes  2. No  77. I Don't Know |  |
| **WORKFORCE** | | | | |
| Gnrl48 | Does your country have healthcare workforce training and capacity building programs – including medical and nursing schools? |  | 1. Yes  2. No  77. I Don't Know |  |
| Gnrl49 | For which specialties and subspecialties in cancer care continuum does the country have an accredited training opportunity (including residency and fellowship)? | 1. Anatomic pathology  2. Anesthesiology  3. Cardiology  4. Clinical Pharmacy  5. Gynecology  6. Gynae-oncology  7. Intensive Care  8. Interventional Radiology  9. Medical/clinical oncology  10. Neuro-Oncology  11. Nuclear Medicine  12. Nursing  13. Nutrition/Dietician  14. Palliative care  15. Physical/Occupational/Rehabilitation Therapy  16. Psychiatry  17. Psychology  18. Pulmonary Medicine  19. Radiation Oncology  20. Radiology  21. Respiratory Therapy  22. General surgery  23. Surgical Oncology  24. Medical Physics  25. Cervical Ablation treatment training  26. Cytotechnology  27. Colposcopy  28. Others | 1. Residency Training Program (within country)  2. Residency Training Program (nearby country)  3. Fellowship Training Program (within country)  4. Fellowship Training Program (nearby country)  77. I don’t know |  |
| Gnrl50 | What are the sources of funding for training and capacity building of health personnel, including medical and nursing schools, continuing education, etc.? Select all that apply: |  | 1. Central Government/Ministry  2. Private donors  3. Multilateral organizations  4. NGO  5. Religious Organizations  6. Student Fees  7. Public-Private Partnerships  8. International Scholarships  77. I Don't Know  **[select]** |  |
| Gnrl51 | Is there a shortage of health professionals in your country? | Anatomic pathology  2. Anesthesiology  3. Cardiology  4. Clinical Pharmacy  5. Gynecology  6. Gynae-oncology  7. Intensive Care  8. Interventional Radiology  9. Medical/clinical oncology  10. Neuro-Oncology  11. Nuclear Medicine  12. Nursing  13. Nutrition/Dietician  14. Palliative care  15. Physical/Occupational/Rehabilitation Therapy  16. Psychiatry  17. Psychology  18. Pulmonary Medicine  19. Radiation Oncology  20. Radiology  21. Respiratory Therapy  22. General surgery  23. Surgical Oncology  24. Medical Physics  25. Cervical Ablation treatment training  26. Cytotechnology  27. Colposcopy  28. Others | 1. Yes  2. No  77. I Don't Know |  |
| Gnrl52 | What efforts, if any, have been made in recent years to expand workforce training capacity or alter the content of training to address shortages? | 1.Developing Specialized Training Programs for the Oncology Workforce  2. Collaboration with academic institutions to improve training opportunities  3. Implementation of continuing education programs for healthcare professionals  4. Adoption of online training platforms for distance learning  5. Partnering with international organizations to provide training support (please specify)  6. Introducing incentives to attract and retain oncology healthcare professionals  7. Enhancing interdisciplinary training to promote teamwork and collaboration  8. Other (please specify) |  |  |
| Gnrl53 | Is specialized training in advanced medical education free to the student (i.e., residency/specialty training)? | 1. Faculty of Medicine  2. School of Nursing  3. Mental Health/Psychology Training  4. Physical Therapy/Rehabilitation Training  5. Radiology Technician/Technologist Training  6. School of Pharmacy  7. Graduate Medical Education (Residency) | 1. Yes  2. No  77. I Don't Know |  |
| Gnrl54 | Are there any unused or underutilized radiology services/equipment in your country due to a shortage of trained personnel? |  | 1. Yes  2. No  77. I Don't Know |  |
| Gnrl54 | Are there any unused or underutilized radiology services/equipment in your country due to a shortage of trained personnel? |  | 1. Yes  2. No  77. I Don't Know |  |
| Gnrl55 | If yes, which radiology machines are not being used due to a shortage of trained personnel? |  | 1. Conventional X-rays  2. Fluoroscopy Unit  3. Ultrasonography  4. Mammography Machines  5. CT  6. Magnetic Resonance Imaging  7. PET  8. Gamma (SPECT) and (SPECT/CT)  9. Interventional Radiology (I.R.) Suite  11. Other (please specify): |  |

| **ID** | **INFO SYSTEMS Question text** | **Sub-question text** | **Answer options** | **Enable conditions** |
| --- | --- | --- | --- | --- |
| Syst1a | Is there a system for registering vital statistics? | If so, please provide the name of the system or Institution/Unit responsible | 1. Yes  2. No  77. I Don't Know |  |
| Syst1b | Is vital statistics data captured in an encoded/electronic format? |  | 1. Yes [insert name of institution/unit in charge of this task]  2. No  77. I Don't Know | If Syst1a=1 |
| Syst1c | Is mortality data available electronically and accessible to the cancer registry? |  | 1. Yes [please provide available data from last year]  2. No  77. I Don't Know | If Syst1b=1 |
| Syst1d | Is there a unique national identification number for each citizen/resident of the country? |  | 1. Yes, they all have a unique national identification number assigned to them  2. Yes, currently in the process of implementation, with partial coverage  3. Implementation is currently being planned  4. No  77. I Don't Know |  |
| Syst2 | Is there a periodic census collecting data on population? | If so, | 1. Yes [Please provide last year it was carried out]  2. No  77. I Don't Know |  |
| Syst3 | Please specify the information relevant to cervical cancer control collected in the census, if any: |  | 1. Screening status (e.g., up-to-date, pending)  2. History of benign/premalignant lesions  3. History of cancer and treatment  4. No Information Collected  5. Other Cancer-Related Information Collected (Explain) |  |
| Syst4 | Does a standardized system for recording patient level data at primary care public facilities exist? |  | 1. Yes  2. No  77. I Don't Know |  |
| Syst5 | What type of system is it? |  | 1. Paper-based  2. Electronic  3. Mixed  4. Other (specify)  77. I Don't Know |  |
| Syst6 | What is the scope of the system? |  | 1. National (the entire population of the country)  2. Subnational (covers defined regions or specific segments of the population)  3. Other (please specify)  77. I Don't Know |  |
| Syst7 | Please specify which data items are collected through the primary care information system, if any: |  | 1. Screening status  2. Date of last screening  3. Reported symptoms or reasons for consulting  4. Record of referral for suspected cancer  5. Cancer diagnosis  6. Cancer treatment |  |
| Syst8 | What is the proportion of the system's target population covered? |  | 1. 1-25%  2. 26-50%  3. 51-75%  4. 76% or more  77. I Don't Know |  |
| Syst9 | Regarding the feasibility of using primary care data for cancer surveillance, please provide the following information: | 1. Is primary care data collection/monitoring for surveillance permitted by current national data regulations?  2. Does the current information system allow for individual-level data linkage with other data sources? | 1. Yes  2. No  77. I Don't Know |  |
| Syst10 | Does a standardized system for recording patient level data at secondary-tertiary care public facilities exist? |  | 1. Yes  2. No  77. I Don't Know |  |
| Syst11 | What type of system is it? |  | 1. Paper-based  2. Electronic  3. Mixed  4. Other (specify)  77. I Don't Know |  |
| Syst12 | What is the scope of the system? |  | 1. National (the entire population of the country)  2. Subnational (covers defined regions or specific segments of the population)  3. Other (please specify)  77. I Don't Know |  |
| Syst13 | What is the proportion of the system's target population covered? |  | 1. 1-25%  2. 26-50%  3. 51-75%  4. 76% or more  77. I Don't Know |  |
| Syst14 | Regarding the feasibility of using secondary/tertiary care data for cancer surveillance, please provide the following information: | 1. Is secondary/tertiary care data collection/monitoring for surveillance permitted by current national data regulations?  2. Does the current information system allow for individual-level data linkage with other data sources? | 1. Yes  2. No  77. I Don't Know |  |
| Syst15. | Is there a legal framework supporting mandatory reporting of cancer cases to a given institution or database? |  | 1. Yes [Please add comments on how that is enforced]  2. No  77. I Don't Know |  |
| Population based cancer registries | | | | |
| Syst16 | How many population-based* cancer registries are there in your country?  *: A population-based registry covers a defined population within country or geographically defined region, aiming to capture all cancers in that population. |  | 1. 0  2. 1  3. 2  4. 3  5. 4  6. 5  7. 6 or more  77. Don’t know |  |
| Syst17 | Registry information: | 1. Registry Name:  2. Responsible/Partner  2. Contact (email, telephone, etc):  Year in which registry started |  | One set of questions for each registry |
| Syst18 | Please mark which of the following cancers are covered by the registry: | 1. All cancers  2. Cervical  3. Other cancers |  |  |
| Syst19 | Coverage of target population |  | 1. Population covered %  2. Please add details on how this figure was estimated (data sources and method): |  |
| Syst20a | Cervical Cancer Incidence Estimated by Cancer Registry |  | 1. Number of cases  2. Rate per 100,000 |  |
| Syst20b | Most recent year of incidence available |  | [list] |  |
| Syst20c | Cervical Cancer Mortality Estimated by Cancer Registry |  | 1. Number of cases  2. Rate per 100,000 |  |
| Syst20d | Most recent year of mortality available |  | [list] |  |
| Syst21 | Please mark the main sources of information for the registry: |  | 1. Public Primary Care Services  2. Private Primary Care Services  3. Public Secondary/Tertiary Care Services  4. Private Secondary/Tertiary Care Services  .5. Public Pathology laboratories  6. Private Pathology laboratories  7. Hospices  8. Death certificates  9. Death registration / Vital Statistics databases  10. Other (please specify): |  |
| Syst22 | Number of full-time equivalent staff |  | [number] |  |
| Syst23 | Are other conditions monitored by registry staff (e.g. other NCDs, such as diabetes; or communicable diseases such as respiratory viruses)? |  | 1. Yes (specify which other medical conditions are monitored by registry staff)  2. No  77. I Don't Know |  |
| Syst24 | Which Information System or Software is used for registration? |  |  |  |
| Syst25 | Is case ascertainment any of the following: |  | 1. Active 2. Passive 3. Other 4. I don’t know |  |
| Syst26 | Does the registry produce periodic reports? | Yes  No | 1. Yes, annual reports  2. Yes, every 2-3 years  3. Yes, every 4-5 years  4. No, reports are produced on an ad-hoc basis  5. No, reports usually don't happen  6. Other (please specify):  77. I Don't Know |  |
| FACILITY-BASED CANCER REGISTRY | | | | |
| Syst27 | How many hospital-based cancer registries are there in your country? |  |  |  |
| Syst28 | Registry information: | 1. Registry Name:  2. Responsible/Partner Institution(s):  3. Contact (email, telephone, etc): |  |  |
| Syst29 | Please mark which of the following cancers are covered by the registry: | 1. All cancers  2. Cervical  3. Other cancers |  |  |
| Syst30 | Indicate the number (average or estimated) of cervical cancer patients registered for the first time annually |  |  |  |
| Syst31 | From the following indicators, please select those that are collected and estimated with information collected in the registry, for patients with cervical cancer | 1. National Identification Number 2. Hospital Record Number 3. Date of admission (admission) 4. Date of Discharge 5. Origin (e.g., primary care service, specialty, screening program, etc.) 6. Comorbidity (pre-existing conditions) 7. Diagnostic studies (e.g., biopsy, histopathology, etc.) 8. Staging studies (e.g., tomography, MRI, ultrasound, PET-CT, etc.) 9. Staging (TNM, tumor size, no. nodules examined, no. nodules positive, distant metastases) 10. Treatment Start Date 11. Surgical treatment (type, extent, etc.) 12. Post-surgical pathology (morphology, surgical margins, residual disease, parametrial involvement, etc.) 13. Radiation therapy (teletherapy or brachytherapy, protocol, dosage, etc.) 14. Chemotherapy (drugs, protocol, etc.) 15. Treatment Complications 16. Contact information (phone, mail, address, etc.) 17. Clinical follow-up information (date and type of consultation, service, etc.) 18. Other information (e.g. for administrative purposes) | [select] |  |
| Syst32 | Please indicate the main sources of information for the registry within the facility: | 1. Accident and Emergency Department  2. Medical Oncology Department  3. Hematology Department  4. Surgical Oncology Department  5. Pathology Department  6. Geriatrics Department  7. Radiology Department  8. Other (please specify): |  |  |
| Syst33 | Please select the main sources of information for the registry outside the facility: | 1. Primary care facilities  2. Hospices  3. Out-patient care specialized facilities  4. Other Secondary/Tertiary Care facilities  5. Private facilities  6. Civil/death registration  7. Other (please specify): |  |  |
| Syst34 | How many full-time equivalent staff work for the facility-based registry? |  |  |  |
| Syst35 | Are other conditions monitored by registry staff or persons in charge of data collection for cancer registration at the facility (e.g. other NCDs, such as diabetes; or communicable diseases such as respiratory viruses)? |  | 1. Yes (specify which other medical conditions are monitored by registry staff)  2. No  77. I Don't Know |  |
| Syst36 | Information System or Software used for registration |  |  |  |
| Syst37 | Does the registry produce periodic reports? If so, how often |  | 1. Yes, annual reports  2. Yes, every 2-3 years  3. Yes, every 4-5 years  4. No, reports are produced on an ad-hoc basis  5. No, reports usually don't happen  6. Other (please specify):  77. I Don't Know |  |
| Syst38 | How does the information from the registry, including periodic reports if any, are used to assess the quality of care and/or influence patient care? | [select all relevant answers] | 1. Monitoring of adherence to management guidelines  2. Monitoring of timeliness of tests, interventions and other services  3. Assessing patient volumes  4. Assessment of availability of resources and needs to meet demands  5. Assessment of patient outcomes: perioperative complications  6. Assessment of patient outcomes: perioperative mortality  7. Assessment of clinical staff workload  8. Assessment of clinical staff performance in terms of patient outcomes  9. Please add information on any additional indicators monitored with registry information |  |
| Syst39 | H.1.o) Regarding the feasibility of using secondary/tertiary care data for cancer surveillance, please provide the following information: | 1. Is secondary/tertiary care data collection/monitoring for surveillance permitted by current national data regulations?  2. Does the current information system allow for individual-level data linkage with other data sources? | 1. Yes  2. No  77. I Don't Know |  |
| **Screening registry** | | | | |
| Syst40 | Are there any screening registries* in your country?*Screening registries are information systems that collect, store and use cancer screening data on individual basis for program monitoring, evaluation and reporting |  | 1. Yes  2. No  77. I Don't Know | One page for each registry |
| Syst41 | How many screening registries are there in your country? |  | 1. 1  2. 2  3. 3 or more  77. I Don't Know |  |
| Syst42 | Please provide the following information for screening registry 1: |  | 1. Name of Screening Registry  2. Responsible/ Partner Institution(s) |  |
| Syst43 | Is this screening registry population-based or hospital-based? |  | 1. Population-based (geographically defined)  2. Facility-based  77. I Don't Know |  |
| Syst44 | What are the main sources of information for this screening registry? |  | 1. Public Primary Care Services  2. Private Primary Care Services  3. Public Secondary/Tertiary Care Services  4. Private Secondary/Tertiary Care Services  5. Public Pathology laboratories  6. Private Pathology laboratories  7. Other (please specify): |  |
| Syst45 | Information System or Software used for registration |  |  |  |
| Syst46 | S.1.e) How many full-time equivalent staff work for the cancer screening registry? |  | 1. 0  2. 1  3. 2  4. 3  5. 4  6. 5 or more  77. I don’t know |  |
| Syst47 | S.1.f) Are conditions other than breast and cervical cancer monitored by registry personnel in your screening database? |  | 1. Yes (specify which other medical conditions are monitored by registry staff)  2. No  77. I Don't Know |  |
| Syst48 | S.1.g) Does the screening database publish period reports? |  | 1. Yes, annual reports  2. Yes, every 2-3 years  3. Yes, every 4-5 years  4. No, reports are produced on an ad-hoc basis  5. No, reports usually don't happen  6. Other (please specify):  77. I Don't Know |  |
| **Treatment registry/clinical audits** | | | | |
| Syst49 | Is there any clinical registries (also called specialized clinical audits) associated with the cervical program treatment services in the country? |  | 1. Yes  2. No  77. I Don't Know | One page for each registry |
| Syst50 | Please provide the following information on any treatment registries or specialized clinical audit databases that collect and monitor treatment information for breast and/or cervical cancer: |  | 1. Name of Registry  2. Responsible/Partner Institution(s)   3. Population- or Hospital-based   4. Coverage (%) of target population   5. Number of FTE staff in registry  6. Are other conditions monitored by registry staff (Y/N)  7. Main sources of information  8. Includes breast and cervical cancer (Y/N)  9. Publishes periodic reports (Y/N)  10. Name of information system/software used for registration |  |
|  |  |  |  |  |

| **ID** | **CERVICAL CANCER QUESTIONS** | **Sub-question text** | **Answer options** | **Enable conditions** |
| --- | --- | --- | --- | --- |
|  | | | | |
| **CERVICAL CANCER PRIMARY PREVENTION** | | | | |
| Prim1 | Are there any publicly funded campaigns developed for cervical cancer, education and/or information targeted to the general population in the last five years? |  | 1. Yes, funded by the public sector [Please indicate institution(s)]  2. Yes, funded by an NGO, civil society [Indicate]  3. Yes, funded by the private sector [Please indicate]  4. Yes, Other Funds [Please Indicate Institution(s)]  5. No campaigns  77. I don't know |  |
| Prim2 | What methods, if any, are used to disseminate campaign information? | 1. Public campaigns  2. NGO/Civil Society Campaigns  3. Private campaign  4. Other [please specify the institution and nature of the campaign] | 1. Social Media Reach  2. Public Service Announcements in the Media  3. Printed educational materials in health centers  4. Other [Indicate] |  |
| Prim3 | Does the primary care network participate in awareness and health literacy campaigns related to cervical cancer and HPV infection? |  | 1. Yes  2. No  77. I Don't Know |  |
| Prim4 | Are NGOs (non-governmental organizations)/CSOs (civil society organizations) involved in the dissemination of IEC (information, education and communication) for cervical cancer screenings, treatment, and palliative interventions? |  | 1. Yes  2. No  Please provide details of these information, education and/or communication campaigns, if any  77. I don't know |  |
| **HPV VACCINATION** | | | | |
| Prim6 | Is immunization against HPV part of the national immunization schedule? |  | 1. Yes  2. No  77. I Don't Know |  |
| Prim6 | Please indicate the year in which the HPV vaccine became available in the public and in the private health sector: | 1. Public  2. Private | [Select year] |  |
| Prim7 | Please indicate the age range(s) of the target population of the national HPV immunization program: | 1. Girls  2. Boys | From age to age |  |
| Prim8 | Where is the national immunization HPV vaccine generally provided? |  | 1. Community  2. School  3. Primary care  4. Secondary/Tertiary Care  5. Other (specify)  77. I don’t know |  |
| Prim9 | Number of doses needed for a complete schedule: | 1. Girls  2. Boys | 1. 1 dose  2. 2 doses  3. 3 doses  4. Provide information on other schedules for special populations, if any:  77. I don't know |  |
| Prim10 | Indicate the percentage of coverage of the target population, separately for boys and girls, for the latest year available: |  | 1. Target population of girls covered %  2. Target population of children covered %  3. Indicate the year of coverage, data sources, and estimation method |  |
| Prim11 | Are there any other important HPV vaccination providers, besides the national immunization program? |  | 1. Private primary care facilities  2. Private secondary care facilities  3. Private Pharmacies  4. NGO initiatives or campaigns  5. Other (please specify): |  |
| Prim12 | Please indicate the most frequent financial cover of the HPV vaccine: |  | 1. Free at the point of use  2. Reimbursement  3. Co-payment  4. Mostly out of pocket  77. I don't know |  |
| Prim13 | Does the country have a registry that captures and monitors individual-level information HPV immunization (e.g. list of eligible individuals, track of received and pending doses, etc.)? |  | 1. Yes (specify)  2. No  77. I Don't Know |  |
| Prim13b | How often is information on HPV vaccination coverage generally shared using the UNICEF/WHO Joint Report Form? |  | 1. Four months after the end of the year (April)  2. Six months after the end of the year (June)  3. 1-2 years after the reported year-end  4. More than 2 years after the reported year-end  5. Information is shared irregularly  6. No Information Sharing  7. I don't know |  |
| Prim14 | Was there any of the following disruptions to the HPV immunization program due to the COVID-19 pandemic? |  | 1. School closures  2. Limited access to health care facilities  3. Social distancing and movement restrictive measures  4. Vaccine shortage at national/regional level  5. Reallocation of public health financial resources  6. Reallocation of public health human resources  7. No disruptions to HPV vaccination efforts  8. Other (please specify) |  |
| Prim15 | If there was a disruption to the HPV immunization program due to COVID-19, what was the estimated percentage of reduction in coverage? | Please provide details on how this reduction was estimated (data sources, calculation method): | 1. No reduction  2. 1-25%  3. 26-50%  4. 51-75%  5. More than 75%  77. I don't know |  |
| Prim16 | Are there still remaining disruptions initially brought upon by the COVID-19 pandemic in any of the following: |  | 1. Reduction in HPV vaccination coverage  2. Limited access to health care facilities  3. Social distancing and movement restrictive measures  4. Vaccine shortage at national/regional level  5. Reallocation of public health financial resources  6. Reallocation of public health human resources  77. I don't know |  |
| **CERVICAL CANCER SCREENING** | | | | |
| Sec1 | Please select the main initial screening test for cervical cancer and premalignant lesions: |  | 1. Cytology  2. HPV test  3. Cytology and HPV co-testing  4. Visual Inspection with Acetic Acid  5. Other (please specify) |  |
| Sec2a | Indicate the age range(s) of the population eligible for **cytology** through the cervical cancer program |  | From age ___ to age ____ |  |
| Sec2b | Indicate the age range(s) of the population eligible for the **HPV test**  through the cervical cancer program |  | From age ___ to age ____ |  |
| Sec2c | Indicate the age range(s) of the population of HIV-positive or immunocompromised women eligible for **HPV testing** through the cervical cancer program |  | From age ___ to age ____ |  |
| Sec2d | Indicate the age range(s) of the population eligible for **visual inspection with acetic acid (VIA)** by the cervical cancer program |  | From age ___ to age ____ |  |
| Sec3 | How many women are eligible for the initial cervical cancer screening test per annum? |  |  |  |
| Sec4 | How is the number of women eligible for cervical cancer screening estimated (data sources, method)? |  |  |  |
| Sec5 | How do women generally access initial screening tests of the cervical screening program? | 1. Cytology  2. HPV test – taken by provider  3. HPV test – self-sampling  4. Visual Inspection with Acetic Acid  5. Other (please specify) | 1. Test available to eligible women who request it at public health facilities  2. The test is offered to eligible women when they are in contact with general health services  3. The test is offered to eligible women when they are in contact with health services for specific conditions (e.g., HIV services, perinatal care, etc.) [Please specify]  4. Phone call from a health worker/community navigator  5. Home visit of the health worker/community navigator  6. Personalized text invitation when eligible/due  7. Personalized mail invitation when eligible/due  8. Mainly through private services  9. Other [specify]:  77. I don't know |  |
| Sec6 | In which of the following settings do women generally access cervical screening program screening? | 1. Cytology  2. HPV testing – taken by provider  3. HPV test – self-sampling  4. IVAA  5. Other [Specify]  [Select the appropriate test(s)] | 1. Primary Care: Home Visit  2. Primary care: community campaigns  3. Primary care: local clinic  4. Primary care: private  5. Mid-level clinic: public  6. Mid-level clinic: private  7. Specialized public center  8. Mostly private  9. Other [specify] |  |
| Sec7a | How many public health centers offer screening services and what percentage of the eligible population has access to screening services within their health center catchment area? | 1. Cytology  2. HPV testing – taken by provider  3. HPV test – self-sampling  4. IVAA  5. Other [Indicate]  [Select the appropriate test(s)] | Primary care:  1. [Indicate number of installations]  2. [Indicate approx. coverage]  Intermediate level care:  1. [Indicate number of installations]  2. [Indicate approx. coverage]  Specialized care:  1. [Indicate number of installations]  2. [Indicate Approximate Coverage]  77. I don't know |  |
| Sec7b | Please indicate how many machines of each of the WHO-prequalified HPV testing systems does the country have for its screening activities? | 1. CareHPV  2. Abbott  3. Cobas 5800  4. Xpert  5. Other: | [Specify number of machines of each brand] |  |
| Sec8 | Please indicate if and which provinces, regions, districts, or particular populations have limited/insufficient access to cervical screening | 1. Cytology  2. HPV testing – taken by provider  3. HPV test – self-sampling  4. IVAA |  |  |
| Sec9a | Is there a standard operating procedure for sample referral* or an integrated specimen referral system?  *Process for bringing screening samples to the lab and reporting results | 1. Cytology  2. HPV testing – taken by provider  3. HPV test – self-collection  4. IVAA  5. Other [Indicate]  [Select the relevant test(s)] | 1. Yes  2. No  77. I don't know |  |
| Sec9b | Please attach the documents (up to 4 files) with the standard operating procedures for sample reference and sample reference form, if any. | [Attach files] |  |  |
| Sec10 | Please indicate the most frequent financial cover of the initial cervical cancer screening test:  Please provide the percentage of coverage of the target population, for the latest available year | 1. Cytology  2. HPV testing – taken by provider  3. HPV test – self-collection  4. VIAA  5. Other [Specify]  [Select the appropriate test(s)] | 1. Free at the point of use  2. Reimbursement  3. Co-payment  4. Mostly out of pocket  77. I don't know |  |
| Sec11 | Indicate the number of women in the target population who underwent screening in the last available year | 1. Cytology  2. HPV testing – taken by provider  3. HPV test – self-collection  4. VIAA  5. Other [Specify]  [Select the appropriate test(s)] | 1. Target population covered (in number of women)  2. Specify the year of coverage, data sources, and estimation method  77. I don't know |  |
| Sec12 | Has there been a reduction in the coverage of cervical cancer screening due to the COVID-19 pandemic? | Please provide details on how this reduction was estimated (data sources, calculation method): | 1. No reduction  2. 1-25% reduction  3. 26-50% reduction  4. 51-75% reduction  5. More than 75% reduction  77. I don't know |  |
| Sec13 | Is there a standard protocol for following up and managing women with a positive initial screening test for cervical cancer and pre-malignant lesions? | 1. High-risk or premalignant lesions  2. Suspected cancer or invasive cancer | 1. Yes  2. No  77. I don't know |  |
| Sec14 | Please indicate the follow-up test after a positive initial screening test for cervical cancer: | Select the initial test that is being conducted in the country:  1. Cytology  2. VIAA  3. HPV DNA Test  4. HPV Genotyping  5. Other [Specify] | 1. Cytology  2. IVAA  3. HPV DNA Test  4. HPV Genotyping  5. Colposcopy  6. Thermal ablation  7. Other [Specify]  77. I don't know |  |
| Sec15 | What is the approximate proportion between HPV, VIA and cytology that is performed annually? | Please add percentages that add up to 100% of women for  1. Screening Test  2. Follow-up test | 1. Cytology  2. HPV testing – taken by provider  3. HPV test – self-sampling  4. VIAA  5. Other [Indicate]  77. I don't know |  |
| Sec16 | Is there any type of treatment available in the centers where cervical screening is performed? | 1. Thermal ablation  2. Cryotherapy  3. Other [Specify] | 1. Yes, >50%  2. Yes, 20-50%  3. Yes, <20%  4. No  77. I don't know |  |
| Sec17 | Select the management/treatment algorithms that are used in your country: | 1. VIAA – Ablation – LLETZ (Screen and treat)  2. HPV DNA – Ablation – LLETZ (Screen and treat)  3. Cytology – HPV DNA – Colposcopy – Management based on colposcopy or histopathology  4. HPV 16/18 – Ablation – LLETZ (Screen and treat)  5. HPV DNA – IVA Triage – Ablation – LLETZ  6. HPV DNA – Colposcopy – Management based on colposcopy or histopathology  7. HPV DNA – Cytology – Colposcopy  8. HPV DNA – Colposcopy – Biopsy and management according to histopathological result  9. Other [Specify] |  | If Sec16=1 |
| Sec18 | List the most common financial coverage for the follow-up test for women who had a positive screening test |  | 1. Free at the point of use  2. Reimbursement  3. Co-payment  4. Out-of-pocket expense  77. I don't know |  |
| Sec19 | Are there waiting time standards for any of the follow-up activities after a positive screening test? | 1. Initial test to result reported to care providers  2. Initial test to patient notification  3. Patient notification to referral for follow-up test  4. Patient notification to follow-up test  5. Follow-up test to histopathological diagnosis confirmation  6. Follow-up test to diagnosis exclusion  7. Diagnosis to treatment initiation: Thermal ablation  8. Diagnosis at the start of treatment: LLETZ  9. Diagnosis at the start of treatment: Other, please specify | 1. Yes  2. No  77. I don't know |  |
| Sec20 | After a positive initial screening test, what is the average time between the following steps? | 1. Initial test to result reported to care providers  2. Initial test to patient notification  3. Patient notification to referral for follow-up test  4. Patient notification to follow-up test  5. Follow-up test to histopathological diagnosis confirmation  6. Follow-up test to diagnosis exclusion  7. Diagnosis to treatment initiation: Thermal ablation  8. Diagnosis at the start of treatment: LLETZ  9. Diagnosis at the start of treatment: Other, please specify | 1. Less than 2 weeks  2. 2-3 weeks  3. 4-6 weeks  4. 7-9 weeks  5. 10 weeks or more  77. I don't know |  |
| Sec21 | Has there been a reduction in the coverage/availability of follow-up tests after a positive cervical cancer screening test, due to the COVID-19 pandemic? | Please provide details on how this reduction was estimated (data sources, calculation method): | 1. No reduction  2. 1-25% reduction  3. 26-50% reduction  4. 51-75% reduction  5. More than 75% reduction  77. I don't know |  |
| **CERVICAL CANCER DIAGNOSIS AND TREATMENT** | | | | |
| Crv1 | Regarding the availability of the following categories of equipment/devices to investigate and/or diagnose cervical cancer and pre-cancer (in the public sector) as part of the Cervical Cancer Control Program (or comparable document), please provide the following information: | 1. PCR Machines  2. Colposcopy  3. LLETZ/LEEP  4. Cryotherapy devices  5. Thermal ablation devices | 1. Number  2. Brand(s)  3. Operator profession (i.e. technicians, nurses, etc.)  4. Number of operators available  5. Number of facilities that offer service |  |
| Crv2 | Are there any surveillance or control systems to monitor adherence to national standards (e.g., clinical guidelines) for cervical cancer treatment? | Yes  No  Do not know |  |  |
| Crv3 | Please list the main public providers of histopathology services for the diagnosis of cervical cancer, and their location | 1-5 main centers |  |  |
| Crv4a | Is there a standardized pathology notification form for cervical cancer tissue samples (or premalignant lesions) in your country? | Hospital/Facility Name  Location | 1. Yes  2. No  77. I don't know |  |
| Crv4b | Please attach the standardized pathology notification form for cervical tissue samples, if there is one | [attach form] |  |  |
| **CERVICAL CANCER TREATMENT** | | | | |
| Crv5 | Which of the following modalities to treat cervical cancer are available in the public sector? | 1. Surgery  2. External radiation therapy  3. Brachytherapy  4. Chemotherapy  5. Palliative care | 1. Yes  2. No, only in private centers  3. Not available in the country  77. I don't know |  |
| Crv6 | Please indicate the most frequent financial cover of the different services and treatment options for cervical cancer: | 1. Surgery  2. Chemotheraphy  3. Conventional Radiotherapy  4. Supportive products in hospital (blood products, nutritional support, etc.)  5. Medications (antibiotics, antiemetics, analgestics)  6. Palliative and supportive care services  7. Supportive products for at home care (nutritional support, wound vac, etc.) | 1. Free at the point of use  2. Reimbursement  3. Co-payment  4. Out-of-pocket expense  77. I don't know |  |
| Crv7 | Are there any surveillance or control systems to monitor adherence to national standards (e.g., clinical guidelines) for cervical cancer treatment? |  | 1. Yes  2. No  77. I don't know |  |
| Crv8 | Select which of the following cervical cancer treatment medicines are available in your country |  | 1. Cisplatin  2. Carboplatin  3. Paclitaxel  4. 5-flourouracil  77. I don't know |  |
| Crv9 | Please list the main **public** providers of cervical cancer treatment and indicate the treatment modalities available at each facility: | 1. Hospital/Facility name  2. Location (City, Province)  3. Treatment services offered (surgery, chemotherapy, radiotherapy) |  |  |
| Crv10 | Please list the main **private** providers of cervical cancer treatment and indicate the treatment modalities available at each facility: | 1. Hospital/Facility name  2. Location (City, Province)  3. Treatment services offered (surgery, chemotherapy, radiotherapy) |  |  |
| Crv11 | Please list the main **non-for-profit** providers of cervical cancer treatment and indicate the treatment modalities available at each facility: | 1. Hospital/Facility name  2. Location (City, Province)  3. Treatment services offered (surgery, chemotherapy, radiotherapy) |  |  |
| Crv13 | Which of the following pain management and supportive treatment medications are available for cervical cancer patients in your country? | 1. Immediate-release oral morphine (tablets)  2. Immediate-release oral morphine (syrup)  3. Long-acting oral morphine  4. Injectable morphine  5. Injectable hydromorphone  6. Injectable fentanyl  7. Fentanyl patch  8. Injectable Methadone  9. Ondansetron oral  10. Ondansetron IV  11. Metoclopramide  12. Lorazepam  13. Midazolam  14. Haloperidol  15. Senna  16. Lactulose  17. Ibuprofen  18. Paracetamol (acetaminophen)  19. Amitriptyline  20. Gabapentin  21. Pregabalin  22. Cyclizine  23. Dexamethasone  24. Granisetron  25. Palonosetron  26. Aprepitant Tablets  27. Aprepitant syrup  28. Olanzapine  29. Docusate sodium  30. Macrogol  31. Omeprazole  32. Ranitidine  33. Carbamazepine  34. Phenobarbital  35. Phenytoin  36. Valproic Acid  37. Levetiracetam  38. Fluoxetine  39. Hyoscine Hydrobromide (Scopolamine) | 1. Sí  2. No  77. No sé |  |
| Crv14 | Which of the following palliative care and support measures are available for patients with cervical cancer? | 1. Inpatient Services  2. Hospice (independent inpatient services)  3. Outpatient Services (Outpatient in the Clinic)  4. Primary care-based services  5. Psychosocial support services  6. Home services  7. Devices necessary for home palliative care (suction, nasogastric tube, subcutaneous access, etc.)  8. Intervention of patients and relatives for hygiene and infection control within their homes  9. Non-Pharmacological Pain Management Services  10. Complex symptom management services  11. End-of-Life Services  12. Bereavement Services  13. No Palliative/Supportive Care Services  77. I don't know  [select] |  |  |
